# Toward adaptive therapeutic timing: integration of mechanistic pharmacology and artificial intelligence in precision dosing

**DOI:** 10.3389/fphar.2026.1890846

**Published:** 2026-09-03

**Authors:** Yufeng Gong, Wei Ren, Ziqin Xiong, Chutian Wu, Congyan Tan

**Affiliations:** 1 Pharmacy Department, The First People’s Hospital of Loudi, Loudi, China; 2 Pharmacy Department, The People’s Hospital of Yuqing County, Zunyi, China; 3 Department of Laboratory Medicine, The First People’s Hospital of Loudi, Loudi, China

**Keywords:** artificial intelligence, machine learning, medication timing, PK/PD, reinforcement learning

## Abstract

Artificial intelligence (AI) is reshaping the dosing of medication. It is shifting practice from fixed schedules to flexible timing that better reflects the needs of individual patients. This review synthesizes recent work in which AI identifies improved windows for drug delivery. It spans multiple time horizons, ranging from minute-by-minute infusion control to regimen planning over weeks or months. These approaches integrate population pharmacokinetic modeling with machine learning and reinforcement learning. Each component serves a distinct purpose. Together, they can recommend the dose timing, adjust dosing intervals, and indicate when a planned treatment pause may be appropriate. The models link these recommendations to the physiological rhythms and current disease status of patients, so timing becomes a defined part of the dosing strategy rather than a simple clock-based rule. Recent advances cluster into three directions. First, reinforcement learning supports sequential dosing decisions in long-term therapies, where early choices shape later outcomes. Second, AI enables chronotherapy by aligning drug delivery with daily circadian biology. Third, hybrid models improve exposure prediction, which supports more accurate interval personalization for individual patients. However, key limitations remain. Many models show limited generalizability across clinical settings, and many methods still require rigorous clinical validation. Even with these constraints, the trajectory is consistent. AI is positioned to move therapeutic timing from a static calendar task to an adaptive, patient-centered element of precision medicine.

## 1 Introduction

Many conversations about drug therapy focus on the drug choice. People often ask, “Which medicine should we use?” The same conversations often ignore another factor that can matter just as much. That factor is the timing of each dose a patient gets. Many care plans still follow fixed schedules such as “once daily” (every 24 h) or “every 2 weeks” (every 14 days). These schedules treat the body like a metronome. This approach assumes that every patient’s body runs on the same steady rhythm. Real bodies do not work that way. The condition of the human body changes from hour to hour. The body changes how fast it clears drugs. The body also changes with disease cycles and daily habits. Small changes in sleep, meals, stress, and activity can also move the best treatment window.

High-variation settings make this problem easier to see. Intensive care and pediatrics often involve fast shifts in body functions. A team can follow the standard protocol and still miss the right drug-exposure window. A dose can still arrive “late” or become less useful even when staff follow the schedule carefully (Beijer et al., 2025). Research also shows a broader issue. Many dosing rules that work for large groups do not reliably fit a single person (Raza et al., 2022). This pattern changes how clinicians should think about timing. Timing is not only an issue of convenience. Timing acts as an “invisible drug” because it can raise or lower the effect of the same dose.

Artificial intelligence (AI) offers a practical way to manage timing across different time scales. AI can support decisions that range from seconds to months. AI shifts the main strategy from “fixing problems after they show up” to “planning ahead before the drift happens.” An AI system can do more than just calculate dose size. The system can also suggest changes in timing. The system can advise a team to administer a dose earlier, delay it, or restart it after a pause. The system bases these changes on changing signals from the patient. The text describes this short-time use in infusion control. Real-time signal integration can support steady, self-correcting infusion behavior (Liu et al., 2025). The text also describes long-time use. AI can help design a person’s dosing rhythm over weeks or months. This design aims to reduce the amount of medication a patient needs while still keeping the desired effect stable.

A timing plan has several parts, and each part can change outcomes. The text treats this timing plan as a needed decision frame. The frame includes administration timing, which links doses to events such as surgery, tissue injury, or daily biological cycles. The frame includes the dosing interval, which is important for maintaining a steady drug level and for planning steps to be taken after a missed dose (Xie et al., 2024). The frame also includes the duration of administration, which guides when a team tapers a dose or ends treatment. The frame includes the gap between dosing periods, which covers planned breaks and safe restart plans. The frame also includes circadian rhythm-based dosing, which accepts that the body can respond differently to the same drug at different times of day. The text also provides concrete ways in which AI can help in these parts. AI can help teams measure and compare missed-dose recovery options. AI can help refine antibiotic infusion schedules. AI can help map safer restart steps after a pause.

Circadian rhythm dosing, often called “chronotherapy,” stands out in this framework. The text explains why this idea is significant. Drug absorption and drug effect can increase and decrease with the day–night cycle. The text links these changes to daily cycles in hormone activity, immune activity, and metabolism. The text also points to a tool that can make this approach more personal. Wearable devices can collect daily-life data. AI can use those data to estimate a person’s own “biological-clock time zone.” This estimate can help align dosing with a time window that aims for strong benefits and fewer side effects. The text gives one specific example. During personalized planning for a drug such as voriconazole, integrated AI models can better capture how circadian patterns differ across patients (Shen et al., 2025).

The text also clarifies that this path has real risks. Data can be uneven across groups, and that can cause bias in a model. Algorithms can also behave differently across patient populations. Clinicians also need safety checks that remain reliable over time. These issues are central barriers that require careful work. Nevertheless, the text describes a clear direction. AI is moving beyond a simple tool for reminders. AI is becoming a partner that helps shape the treatment timeline itself. The text describes this change as a move toward a timing system that connects seconds, days, and months into a more flexible life rhythm.

### 1.1 Methodology

**FIGURE 1:**
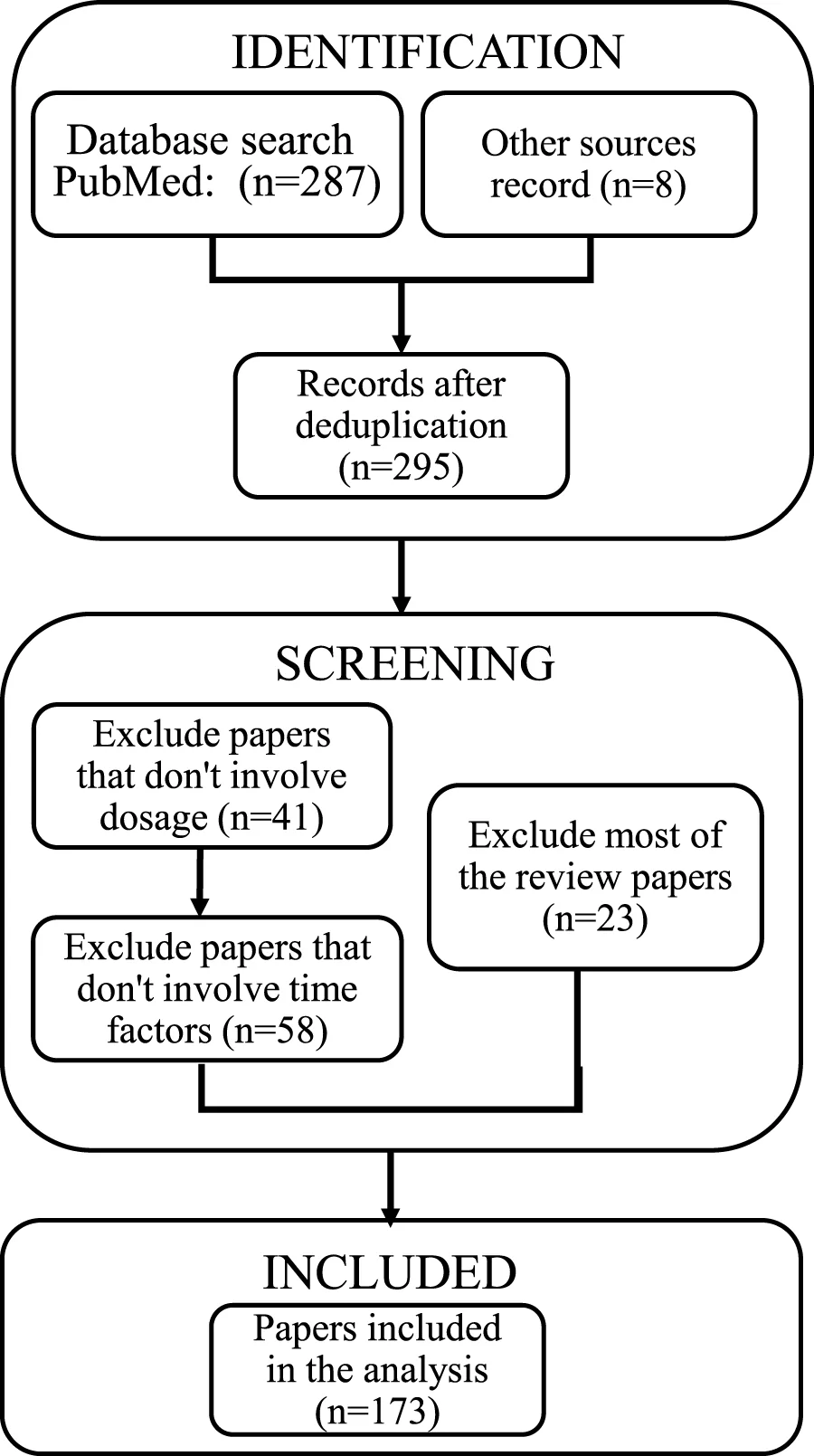
Flowchart for reference screening.

We conducted a review of existing research on the application of AI in the field of medication timing. This review involved a systematic literature search in the PubMed database in September 2025, retrieving nearly all relevant publications concerning AI applications in medication timing. This review focuses on AI applications that have entered the medical/pharmaceutical context rather than the algorithms themselves. Therefore, engineering conference articles were not systematically included. The PubMed database mainly covers areas such as clinical medicine, dentistry, nursing, biomedical sciences, pharmaceuticals, medical history, medical ethics, space, and life sciences, which meets our expected criteria. The publication period was restricted to the past 5 years. The specific search terms were as follows: ((“artificial intelligence” [Title/Abstract] OR “machine learning” [Title/Abstract] OR “deep learning” [Title/Abstract] OR “reinforcement learning” [Title/Abstract] OR “predictive model*” [Title/Abstract] OR “pharmacokinetic model*” [Title/Abstract]) AND (“drug administration schedule” [MeSH Terms] OR “chronotherapy” [MeSH Terms] OR “drug timing” [Title/Abstract] OR “dosing interval*” [Title/Abstract] OR “dose timing” [Title/Abstract] OR “individualized dosing” [Title/Abstract] OR “adaptive dosing” [Title/Abstract])). An initial screening of titles and abstracts was subsequently performed. Following the initial selection, full-text articles were retrieved and reviewed for eligibility. The two-stage inclusion criteria were: (1) studies with full English-language texts, (2) studies describing original research by the authors, and (3) studies concerning the application of AI in the field of medication timing. Therefore, the exclusion criteria included: (1) studies without full English-language texts, (2) studies not describing complete original research, such as reviews or book chapters, and (3) studies merely mentioning AI without its actual application. A systematic electronic search yielded a total of 287 records. In addition, eight records previously known by the authors were also considered. After screening according to the inclusion criteria and reviewing the titles, abstracts, and full texts, 173 studies were ultimately selected for the final analysis. The specific screening process is shown in Figure 1.

We have compiled the literature on the main mechanistic pharmacology and artificial intelligence models involved in this study in Table 1.

**TABLE 1:** Causal/mechanism-driven models (PBPK/PopPK/MIPD).

Ref	Drug/Background	Key points	References
1	Vancomycin (neonates)	PBPK is superior for initial dosing; PopPK is better for Bayesian updating	Cao et al. (2025)
2	Natalizumab (MS)	Bayesian MIPD enables interval extension up to 7 weeks	Van Den Berg et al. (2025)
3	Cefotaxime (neonates)	PBPK-derived q6h–q8h regimens by GA/PNA	Li Q. et al. (2024)
4	Infliximab (IBD)	Model selection affects individualized dose recommendations	Chaiben et al. (2025)
5	Aripiprazole LAI	Simulates missed/delayed doses and 2-month interval dosing	Wang et al. (2024)
6	Ganciclovir (lung transplant)	Covariate-driven individualized dosing nomograms	Dvořáčková et al. (2022)
7	Atomoxetine (ADHD)	CYP2D6-driven exposure variability modeling	Cheng et al. (2024)
8	Propylthiouracil	PBPK extrapolated to pediatric/elderly dosing	Shen et al. (2022)
9	Tacrolimus (transplant)	Bayesian dose optimization in liver transplant	Hou et al. (2025)
10	PEGasparaginase	Individualized dosing via TDM-guided PK modeling	Kloos et al. (2020)
11	Insulin icodec	Weekly dosing supported by PK–PD modeling	Pieber et al. (2023)
12	Cefepime (ICU)	Continuous infusion reduces neurotoxicity	Bilal et al. (2023)
13	Meropenem (CKRT pediatrics)	Continuous infusion only regimen meeting targets	Butragueño-Laiseca et al. (2024)
14	Amikacin (neonates)	Simplified vs. complex dosing schemes	Van Der Veer et al. (2025)
15	Nusinersen (SMA)	Defines restart timing after dosing interruption	MacCannell et al. (2022)
16	Atezolizumab	Interval extension maintains exposure and lowers cost	Kicken et al. (2024)
17	Risperidone LAI	q1m vs. q2m interval comparison	Perlstein et al. (2025)
18	Remdesivir	Dosing simulations across regimens	Roberts et al. (2025)
19	Vancomycin (sampling)	Optimal sampling timing improves dosing	Downes et al. (2025)
20	Selumetinib	Compares q6h/q8h/q12h dosing schedules	Köllő et al. (2026)
21	Brexpiprazole (obesity)	Adjusted titration and temporary BID dosing shortens time to effective concentration in obese patients	Bruno et al. (2022)
22	Topiramate (missed doses)	Simulation-guided remedial dosing after missed doses	Methaneethorn and Charoenchokthavee (2022)
23	Oxycodone + ritonavir	Dose or interval adjustment mitigates DDI-related overexposure	Zheng et al. (2024)
24	Isavuconazole (ICU)	Adaptive dosing improves early exposure attainment	Jansen et al. (2023)
25	Meropenem/Piperacillin (ICU)	Early concentration-based adjustment needed during the first interval	Beijer et al. (2025)
26	Vancomycin (PICU)	Higher-frequency dosing improves AUC/MIC target attainment	Sridharan et al. (2021)
27	Alemtuzumab (transplant)	Lean bodyweight-adjusted dosing reduces exposure variability	Zwart et al. (2023)
28	Linezolid (elderly)	Renal function-guided interval/dose reduction lowers toxicity	Chen et al. (2025)
29	Factor VIII (hemophilia A)	Bayesian-guided individualized prophylaxis dosing	He et al. (2023)
30	IVIG (MMN)	More frequent dosing stabilizes IgG levels and symptoms	Li Z. et al. (2024)
31	Hydroxychloroquine (COVID-19)	Compared alternative loading and maintenance regimens	Thémans et al. (2021)
32	Nonadherence modeling	Regimen design robust to missed or delayed doses	Counterman and Lawley (2022)
33	Cefotaxime (neonatal sepsis)	BID dosing achieves PK/PD target in neonates	Shang et al. (2022)
34	GC1118 antibody	Weekly dosing superior to biweekly dosing at equal exposure	Chung et al. (2021)
35	Ceftaroline (CNS/soft tissue)	Higher-frequency dosing improves CNS target attainment	Helfer et al. (2022)
36	Avalglucosidase alfa (Pompe)	Weight-based regimen selection equalizes exposure	Tiraboschi et al. (2023)
37	PEGasparaginase	Individualized clearance-based dosing guidelines	Kloos et al. (2023)
38	Vancomycin neonates	Model robustness assessment for MIPD platforms	Hughes et al. (2023)
39	Tacrolimus (HCT)	Continuous-infusion dosing optimized via Bayesian forecasting	Brooks et al. (2021)
40	Golimumab	Q6W interval improves trough response vs. Q8W	Lee et al. (2022)
41	Nonlinear PK theory	Mathematical rules for dose and timing design	Cao et al. (2024)
42	Linezolid (ICU)	Reduced dose/interval lowers thrombocytopenia risk	Qin et al. (2025)
43	Rifampicin–OCP interaction	Monthly rifampicin avoids contraceptive interaction	Radtke et al. (2023)
44	Tranexamic acid	Supports target-controlled infusion strategies	Liu et al. (2025)
45	Cefmetazole surgery	Renal-function-based re-dosing interval optimization	Komatsu et al. (2022)
46	Snake venom PK	Timing of antivenom guided by venom exposure kinetics	Sanhajariya et al. (2021)
47	Clozapine	Once-daily dosing comparable to divided dosing	Kitagawa et al. (2022)
48	Cefiderocol (pneumonia)	Confirms adequacy of renal-adjusted dosing	Kawaguchi et al. (2022)
49	Testosterone undecanoate	Shorter maintenance interval improves trough levels	Pastuszak et al. (2021)
50	Linezolid (MDR-TB, nonadherence)	Remedial dosing for delayed or missed doses	Li R. et al. (2024)
51	Posaconazole (antifungal)	External validation of PopPK models; Bayesian forecasting improves prediction	Huang et al. (2022)
52	Meropenem	Extended infusion improves PTA; dosing is driven by renal function	Boutzoukas et al. (2025)
53	Benzathine penicillin G	Subcutaneous high-dose allows ≥3-month dosing intervals	Kado et al. (2023)
54	Tacrolimus + voriconazole	Voriconazole markedly reduces tacrolimus clearance	Zhao et al. (2024)
55	Cyclosporine + azoles	2-h dose separation minimizes GI interaction	Shiraishi et al. (2025)
56	Metronidazole (neonates)	Supports 6-h dosing in older infants	Randell et al. (2024)
57	Ceftazidime (PICU)	Weight and eGFR guide individualized dosing	Li M. et al. (2024)
58	Melphalan (HCT)	Renal function > obesity for exposure variability	Shah et al. (2022)
59	Busulfan (pediatrics)	Optimized sampling timing for AUC-guided dosing	Ben Hassine et al. (2024)
60	Levetiracetam	Renal function and phenytoin affect clearance	Singkham et al. (2025)
61	Factor VIII	Bleeding risk varies despite trough >1%	Tiede et al. (2020)
62	Pembrolizumab	Q6W dosing equivalent to Q3W dosing	Jones et al. (2022)
63	Paliperidone palmitate	6-month dosing validated via PK bridging	T’jollyn et al. (2024a)
64	Pembrolizumab (breast cancer)	Extended-interval dosing is safe and effective	LeVee et al. (2025)
65	Lacosamide (pregnancy)	Dose increases needed during gestation	Barry et al. (2025)
66	Risankizumab (psoriasis)	Real-time dosing interval personalization	Thomas et al. (2025)
67	Benzylpenicillin (ICU)	Continuous/extended infusion improves target attainment	Shah et al. (2023)
68	Cefepime	Cystatin-C improves early target attainment	Barreto et al. (2025)
69	Levofloxacin	Higher doses needed with high eGFR	Setiawan et al. (2022)
70	Linezolid (ECMO)	8-h dosing improves PK/PD targets	Milaković et al. (2024)
71	Pexidartinib	Meal timing significantly alters exposure	Nakayama et al. (2024)
72	Cabotegravir/Rilpivirine	Older adults have higher trough exposure	Bettonte et al. (2024)
73	Tobramycin	Single-sample TDM is sufficient	Drennan et al. (2021)
74	Gentamicin (neonates)	Extended dosing intervals are recommended	De Hoop-Sommen et al. (2023)
75	Tislelizumab	Q2W–Q6W regimens are equivalent	Rizwan et al. (2025)
76	Infliximab (pediatric IBD)	Covariate-driven PopPK predicts troughs for individualized dosing	Clemente-Bautista et al. (2024)
77	Imipenem (FN, hematologic malignancy)	q6h extended infusion optimized f%T>MIC	Rao et al. (2025)
78	Meropenem (ICU adults)	Continuous infusion improves target attainment	O’Jeanson et al. (2021)
79	TV-46000 (LA risperidone)	Monthly vs. q2-month dosing flexibility validated	Perlstein et al. (2025)
80	Tobramycin (CF adults)	Height-based regimens evaluated for PTA	El Hassani et al. (2022)
81	Meropenem (infants)	Label dosing validated across maturation	Ganguly et al. (2021)
82	Atezolizumab (SC)	Extended intervals (4–6 weeks) reduce cost	Kicken et al. (2024)
83	Glimepiride	Mechanistic twin enables stratified dosing	Elias and König (2025)
84	Vancomycin (critically ill children)	Optimal sampling improves AUC estimation	Downes et al. (2025)
85	Febuxostat	Renal impairment effects on exposure	Xu et al. (2022)
86	Doxycycline (donkeys)	Veterinary PK characterization	Chapuis et al. (2021)
87	ABL001/CTX-009 (oncology)	q2w vs. q3w regimens are equivalent	Na et al. (2024)
88	Meropenem (infants)	Supports neonatal dosing	Ganguly et al. (2021)
89	Febuxostat	Exposure in renal dysfunction	Xu et al. (2022)
90	Meropenem (PICU, CKRT)	Continuous infusion required in low weight	Butragueño-Laiseca et al. (2024)
91	Selumetinib (pediatric NF-1)	Pediatric exposure characterization	Köllő et al. (2026)
92	PSMA-11 + folate	Folate dose and timing affect tumor uptake	Siebinga et al. (2023)
93	Ampicillin-sulbactam (azotemic dogs)	q12h sufficient in renal disease	Wang Z. et al. (2025)
94	Dexamethasone (pediatric airway)	PBPK supports prophylactic dosing	Van Der Heijden et al. (2024)
95	Pentazocine (children)	Safety-driven dosing simulations	Omori et al. (2025)
96	Trofinetide (Rett syndrome)	Confirms approved regimen	Darwish et al. (2024)
97	Nusinersen (SMA restart)	Optimal re-loading after interruptions	MacCannell et al. (2022)
98	Cefazolin (obesity)	Higher dose needed in ≥120 kg	Chung et al. (2023)
99	Islatravir (HIV PrEP, microneedle patch)	PBPK predicts sustained intracellular exposure for 3–12-month dosing via micro-needle array patches	Kinvig et al. (2022)
100	Voriconazole (liver dysfunction)	TBIL-based PopPK supports dose reduction and longer dosing intervals in hepatic impairment	Tang et al. (2021)
101	Quetiapine (cirrhosis, CYP3A4 polymorphism)	PBPK quantifies exposure increase in cirrhosis and CYP3A4*22 carriers; supports major dose reductions	Lee et al. (2025)
102	Nifedipine (pregnancy)	Pregnancy-specific PBPK/PD improves maternal–fetal safety and individualized dosing	Liu et al. (2024)
103	Vancomycin (adult, non-critical)	Comparative evaluation shows only one PopPK calculator accurately predicts trough levels	Huang et al. (2025)
104	Vancomycin (CVVHDF ICU)	PTA simulations identify optimal loading and 12-h maintenance dosing during CVVHDF	Kirwan et al. (2021)
105	Vancomycin (neonates)	Fluid input and diuretics identified as novel covariates influencing clearance	Zhao K. et al. (2025)
106	Hepatic clearance scaling (HCCC)	Individual MPPGL improves *in vivo* hepatic clearance prediction for PBPK models	Zhou J. et al. (2025)
107	Gentamicin (neonates)	Compares dosing intervals (24 h–48 h); highlights major variability in PTA across guidelines	Grzeskowiak et al. (2025)
108	Natalizumab (MS, pregnancy)	PBPK predicts maternal washout time and optimal infant vaccination timing	Zhou P. et al. (2025)
109	Isoniazid	ML predicts C2h for individualized initial dosing	Tang et al. (2024)
110	Valproic acid (elderly)	ML+PPK improves concentration prediction	Ma et al. (2024b)
111	Precision dosing (general)	RL formulates adaptive sequential dosing decisions	Tosca et al. (2024)
112	Vancomycin (neonates)	ML improves dose individualization vs. PopPK	Tang et al. (2023)
113	Imipenem (febrile neutropenia)	Exposure–response guides infusion frequency	Rao et al. (2025)
114	ICU hemodynamics	RL optimizes timing of fluids/vasopressors	Sabounchi et al. (2025)
115	Therapeutic hypothermia	AI-guided multi-target drug prioritization supports chronotherapeutics	Liu et al. (2022)
116	Infliximab (Crohn’s, pediatrics)	RL agent optimizes dose and infusion interval to reach target trough levels	Irie et al. (2025b)
117	Mycophenolate (pediatric renal disease)	Exposure–response ML models guide individualized dosing	Han et al. (2025)
118	Anticoagulation bleeding risk	Time-series DL predicts bleeding risk (no dosing decision)	Shahryari Fard et al. (2024)
119	Atezolizumab (real-world)	ML identifies covariates driving selection of dosing interval	Vora et al. (2024)
120	Irinotecan toxicity	ML predicts toxicity risk and not dosing decisions	Bedon et al. (2022)
121	Mycophenolate (transplant)	Integrated ML–PK framework for individualized dosing	Tsyplakova et al. (2025)
122	Levetiracetam (epilepsy)	Creatinine clearance and hemoglobin level predict exposure where TDM is unavailable	Jiang et al. (2025)

Table 2 shows the logical associations and data integration methods used in the collected literature to link mechanistic model parameters with artificial intelligence.

**TABLE 2:** Logical relationships and data integration methods linking mechanism model parameters to artificial intelligence.

Integration strategy category	Specific feature engineering approach (Quantification of ADME mechanisms)	Advantages	Limitations/applicability	Selected references
Direct use of individual parameters (CL, V) estimated from population pharmacokinetic (PopPK) models as ML inputs	PopPK-derived individual clearance (CL) and volume of distribution (V), obtained via Bayesian posterior estimation, used as features in ML models alongside other clinical covariates to predict drug concentrations	Significantly improves single-algorithm performance (e.g., VPA model *R* ^2^ = 0.74); enables rapid and cost-effective concentration estimation without full sampling (elderly vancomycin model)	Requires prior PopPK modeling and Bayesian estimation; dependent on initial model reliability; applicable only to populations with established PopPK models (e.g., elderly and pediatric)	Ma et al. (2024b)
	Same as above—PopPK-estimated CL and V as inputs, combined with Boruta feature selection, for XGBoost-based voriconazole concentration prediction	External validation *R* ^2^ = 0.75, RMSE = 0.14; feasible with small samples (n = 76) in children <2 years	Requires prior PopPK parameter estimation; limited to pediatric populations; external validation sample size is modest	Shen et al. (2025)
	PopPK parameters (CL, V) included as features in an ensemble model (SVR+LightGBM+CatBoost) for predicting vancomycin concentrations in elderly patients	Test set *R* ^2^ = 0.656; accuracy within ±5 mg/L = 81.82%; outperforms models without PopPK parameters	Requires estimation of individual CL/V; specific to elderly patients; relies on retrospective data	Ma et al. (2024a)
Use of physiological/biochemical covariates reflecting ADME processes (enzyme activity, renal/hepatic function, etc.) as input features	Inclusion of N-acetyltransferase 2 (NAT2) genotype (reflecting acetylation enzyme activity), body weight, ethnicity, and dosing regimen in a CatBoost model to predict 2-h isoniazid concentrations	Real-world validation overall accuracy 93.4%; MAPE reduced by 45.7% and PTA increased by 43.7% versus PopPK models	Requires genotyping; ethnicity used as a proxy for population, not directly quantifying dynamic enzyme fluctuations; NAT2 status must be known beforehand	Tang et al. (2024)
	Use of creatinine clearance (Ccr) and hemoglobin (HGB) as the primary predictors (multiple linear regression, covariate-based) reflecting renal function and metabolic status	Bootstrap-validated stability; simple equation applicable in resource-limited settings without TDM.	Not an ML algorithm (conventional regression); adjusted *R* ^2^ only 0.44; generalizability requires external validation; no direct integration of enzyme activity	Jiang et al. (2025)
	Inclusion of plasma albumin, calcium, and liver/kidney function markers (ALT, AST, and eGFR) as features in a random forest model to predict mycophenolic acid (MPA) exposure, with SHAP for key factor identification	SHAP analysis identified albumin, calcium, and hepatorenal function as key predictors; proposed weight- and albumin-based stratified dosing	Targeted at pediatric immune-mediated nephropathy; requires laboratory tests; does not directly use enzyme or transporter data	Han et al. (2025)
	Use of covariates (creatinine, postnatal age, body weight, etc.) and dosing regimen in a CatBoost model to first predict vancomycin steady-state trough concentration (C0) and then use C0 plus covariates to predict AUC.	External validation accuracy improved by 42.5% versus PopPK model; virtual trial PTA reached 80.3%, far exceeding standard dosing	Does not directly use transporter or enzyme activity parameters; mainly relies on renal function indices; applicable to neonates; requires external validation	Tang et al. (2023)
Generation of training data via PopPK simulation (virtual patients) followed by ML model training	Based on four published adult PopPK models for isoniazid to simulate individual C_2_h data used to train CatBoost	Integrates multiple PopPK models to improve generalizability; simulated data can cover a broad range of population characteristics	Simulation relies on PopPK model assumptions; real-world data validation is still required (performed in the study)	Shen et al. (2025)
	Using a published pediatric PopPK model for infliximab to generate synthetic concentration data for initial XGBoost training and then correcting residuals with real-world data	Combining synthetic and real data overcomes small-sample limitations; ensemble model performance comparable to that of Bayesian methods	Requires a reliable PopPK model; correction step adds complexity; does not directly input enzyme activity parameters	Irie et al. (2025a)
Reinforcement learning (RL) in PopPK/PopPK-PD simulated environments for dose optimization (indirect ADME integration)	Embedding a full PopPK model (including inter-individual variability and assay error) into a deep Q-network (DQN) simulation environment; the agent learns dosing strategies via interaction, with reward based on target concentration range	DQN policy converged; PTA reached 92.9% and 98.4% at fourth and fifth infusions in virtual patients; retrospective real-world validation showed concentrations closer to target for matched doses	Dependent on PopPK model structure and parameters; requires extensive simulation for training; enzyme activity is implicitly embedded via the PK model rather than explicitly input	Irie et al. (2025b)
	Embedding a PopPK-PD model (erdafitinib) into a Q-learning framework; the agent optimizes dose based on individual PK–PD parameters, with reward function balancing efficacy (phosphate level) and safety	Outperforms FDA-approved adaptive dosing rules; improves efficacy and safety by >10%; flexibly adaptable to different patient parameters	Relies on published PopPK–PD models; virtual validation only, not tested in real patients; requires biomarker monitoring	De Carlo et al. (2024)
Use of limited-sampling concentration points (indirectly reflecting individual ADME differences) combined with covariates	Using 2–3 measured concentration points, sampling time deviations, dose, transplant type, age, etc., in an XGBoost model to estimate tacrolimus AUC.	Requires only 2–3 points; relative bias <5%, relative RMSE <10%; outperforms maximum *a posteriori* Bayesian in an independent full-PK dataset.	Requires TDM sampling; cannot be used for initial dose prediction; concentration points implicitly contain ADME information but do not explicitly quantify enzyme or transporter activities	Woillard et al. (2021)

Based on the systematic comparisons presented in Tables 2–4, artificial intelligence methods—particularly gradient-boosted trees and reinforcement learning—demonstrate significant advantages over traditional population pharmacokinetic (PopPK) and clinical baseline models. By combining PopPK parameters with real-world data, artificial intelligence can improve the accuracy of dose predictions and the rate of target achievement, thereby providing efficient decision support for precision medicine.

**TABLE 3:** Methods and classification of clinical sparse data processing.

Mechanism for handling irregular sampling	Assumption about missing data	Advantages in PK prediction	Limitations in PK prediction
Uses mechanistic differential equations (compartmental models) to describe the concentration–time profile; all individual sampling points (regardless of time alignment) contribute to population and individual parameter estimation via the likelihood function. Bayesian feedback combines population priors with sparse individual data, allowing arbitrary sampling times without requiring fixed time points	Assumes missing data are missing at random (MAR) or completely at random (MCAR); the model utilizes all available observations via likelihood maximization and does not perform explicit imputation. Bayesian methods automatically weigh available information without filling missing values	In neonatal vancomycin, Bayesian MIPD improved prediction accuracy by 42.5% over traditional PopPK (Tang et al., 2023)	Highly dependent on correct model structure (compartment number and covariate relationships); misspecification can introduce bias When sampling is extremely sparse (e.g., only one point), individual parameter shrinkage increases, leading to greater predictive uncertainty, though Bayesian methods still provide posterior distributions Computationally intensive, requiring specialized software (e.g., NONMEM); however, MIPD platforms have partially addressed this (e.g., tacrolimus expert system) (Woillard et al., 2021)
		For tacrolimus, an XGBoost model (derived from PopPK) using 2–3 concentrations with actual time deviations as features achieved relative bias <5% and relative RMSE <10% (Woillard et al., 2021)	
		Imipenem PopPK was successfully built from 207 samples from 121 patients (average 1.7 points/patient) to guide dosing (Rao et al., 2025)	
		Mycophenolic acid PopPK utilized 513 concentrations from 171 pediatric patients (average ∼3 points/patient) to guide individualization (Han et al., 2025)	
Models typically use measured concentrations at specific time points (e.g., trough and 2 h concentration) or concentrations at a limited number (2–3) of time points along with sampling time deviations. To tolerate irregular timing, some models incorporate the actual sampling time deviation from nominal times as a feature. However, they fundamentally require each individual to provide the same number of aligned sampling points; samples missing any required time point are usually excluded	Implicitly assumes complete observation (the required time points must exist); missing samples are directly discarded. No imputation techniques are used. The strategy focuses on point estimates and not on reconstructing the full concentration curve	Isoniazid: CatBoost predicted 2-h concentration with real-world accuracy of 93.4% and reduced MAPE by 45.7% versus that with PopPK (Tang et al., 2024)	Strict requirements on sampling times; performance may degrade if clinical sampling deviates from the training time window Cannot leverage sparse data at arbitrary time points; must rely on predefined time points. Cannot output the full concentration–time curve or AUC unless specifically modeled (e.g., tacrolimus uses 2–3 points for AUC, but those points must be available) Missing data handling is simplistic (case deletion), which wastes information when data are already scarce
		Neonatal vancomycin: CatBoost using dosing regimen +9 covariates predicted trough concentrations prior to therapy, with external validation accuracy 42.5% higher than that of PopPK (Tang et al., 2023)	
		Elderly vancomycin: ensemble model (SVR + Light GBM + CatBoost) achieved ±5 mg/L accuracy of 81.82% (Ma et al., 2024a)	
RL agents are trained in complete PopPK simulation environments that can generate virtual concentration trajectories at arbitrary time points (fully observed). For real-world application, individual PK parameters are first obtained via Bayesian updating (from sparse TDM) and then fed into the trained RL agent for decision-making. Thus, handling of real missingness is inherited from the underlying PopPK.	Does not directly deal with real missing data; training data are fully observed simulations. In real scenarios, missingness is addressed by PopPK Bayesian methods	Infliximab (pediatric Crohn’s): DQN policy achieved PTA of 92.9% and 98.4% at fourth and fifth infusions; retrospective validation showed concentrations closer to target when doses matched RL recommendations (Irie et al., 2025b)	Heavily dependent on the quality of the underlying PopPK model; if the model cannot capture individual variability, RL performance is compromised Training and validation are largely virtual; real-world missing patterns may differ from simulation assumptions, requiring additional adaptation
		Erdafitinib (urothelial cancer): Q-learning improved efficacy and safety by >10% over FDA-approved adaptive rules (De Carlo et al., 2024)	

**TABLE 4:** Quantitative analysis indicators and baseline comparisons.

References	Drug/Field	AI algorithm	Dataset size	AI model performance	Baseline model performance	Relative improvement	Baseline type
Tang et al. (2024)	Isoniazid	CatBoost	Simulated data (N = 61) + real-world validation	Overall prediction accuracy 93.4%	PopPK model	45.7% reduction in MAE; target attainment probability increased by 43.7%	PopPK model
Ma et al. (2024b)	Valproic acid (elderly epilepsy)	Ensemble (CatBoost + LightGBM + RF = 7∶2∶1)	Retrospective cohort (Model N = 782, validation set N = 120)	*R* ^2^ = 0.74 (with PPK parameters)	Single-algorithm models (without PPK parameters)	*R* ^2^ improvement (each single algorithm improved significantly after incorporating PPK parameters)	Single ML algorithms
Tang et al. (2023)	Vancomycin (neonates)	CatBoost (C_0_ and AUC models)	N = 1,894, Large neonatal dataset + external validation	C_0_ external validation accuracy 80.3%	PopPK model	42.5% improvement in prediction accuracy; target attainment 80.3% vs. 37.7%–61.5% (standard doses)	PopPK model/international standard dosing
Sabounchi et al. (2025)	Hemodynamic management (post-cardiac surgery AKI prevention)	Reinforcement learning (RL)	MIMIC-IV: 6,643; SICdb: 2,254; MSHS: 846 (total 9,743)	Higher cumulative reward than clinicians	Clinician strategies	Adjusted OR for pAKI: SICdb 0.92 [0.89–0.96], MSHS 0.91 [0.86–0.96]	Clinician strategies
Irie et al. (2025b)	Infliximab (Crohn’s disease)	Deep Q-network (DQN/RL)	1,000 virtual patients + real-world retrospective validation	PTA 92.9% at 4th infusion, 98.4% at 5th	Actual dosing without DQN recommendations	Patients with DQN-concordant doses had trough concentrations significantly closer to target (P < 0.05)	Real-world clinical dosing
Han et al. (2025)	Mycophenolic acid (MPA, pediatric nephropathy)	Random forest (RF)	171 pediatric patients (513 concentrations), 4∶1 train/test split	RF best among 7 ML models (metrics in original)	PopPK model/other 6 ML algorithms	Direct comparison of PopPK vs. ML; SHAP identified albumin, calcium, liver/renal biomarkers as key predictors	PopPK model + other ML algorithms
Shahryari Fard et al. (2024)	Major bleeding prediction during anticoagulation	Ensemble (feed-forward NN + RNN)	2,542 patients (118 major bleeds), 70%/30% train/test	AUC = 0.82; sensitivity 61%; specificity 82%	Six previously developed clinical models (modified), AUC ∼0.60–0.70	Absolute AUC increase of ∼0.12–0.22	Previous clinical risk prediction models
Vora et al. (2024)	Atezolizumab	XGBoost (best performer)	Real-world data (RWD)	PR-AUC used as metric; best handling of imbalanced data	Other ML algorithms	XGBoost outperformed other evaluated algorithms in PR-AUC	Other ML algorithms
Bedon et al. (2022)	Irinotecan (phase-I MTD prediction)	Random forest (RF)	N = 45, phase-I data (cross-validation + two dose-escalation validation cohorts)	CV accuracy 80.4%; validation accuracy 82.0%; MCC = 0.59	Other 4 ML algorithms	RF superior in MCC (0.549) and accuracy (80.4%) over others	Other ML algorithms
Tsyplakova et al. (2025)	Mycophenolic acid (MPA, renal transplant)	Bagging/boosting (ensemble trees)	76 renal transplant patients	Bagging/boosting *R* ^2^ > 0.91	PopPK model	PopPK+ML combined (PopPK *R* ^2^ ≈ 0.81, ML improved *R* ^2^ by ∼0.10)	PopPK model
Jiang et al. (2025)	Levetiracetam	Multiple linear regression (MLR)	130 epilepsy patients (175 steady-state concentrations), Bootstrap = 1,000	Adjusted *R* ^2^ = 0.440	No traditional PK model comparison provided	Baseline not provided (univariate + multivariate regression only)	Not reported
Woillard et al. (2021)	Tacrolimus	XGBoost	b.i.d.: 4,997 AUCs; q.d.: 1,452 AUCs; six full external PK datasets	Test set relative bias <5%, relative RMSE <10%	MAPBE (maximum *a posteriori* Bayesian estimation)	XGBoost outperformed MAPBE in six independent full PK datasets	MAPBE (Bayesian estimation)
Ma et al. (2024a)	Vancomycin (elderly)	Ensemble (SVR + Light GBM + CatBoost = 6∶3∶1)	Retrospective analysis, (383 × 34 (33 features and one target variable (Drug Concentration)) including PPK	*R* ^2^ = 0.656; MAE = 3.458; ±5 mg/L accuracy 81.82%; ±30% accuracy 76.62%	Single-algorithm models without PPK parameters	All algorithms with PPK parameters outperformed those without; final ensemble best	Single ML algorithms without PPK parameters
De Carlo et al. (2024)	Erdafitinib (urothelial carcinoma)	Q-learning (RL)	141 virtual patients (generated from published PopPK-PD model)	RL-optimized regimen improved both efficacy and safety by >10%	FDA-approved dose-adaptive regimen (based on serum phosphate)	>10% improvement in both efficacy and safety	FDA-approved dose-adaptive rule
Choo et al. (2024)	Adverse event reporting completeness	Random forest (RF) + TreeSHAP	132,738 Malaysian reports (2005–2019)	Best feature set for identifying “well-documented” reports	No conventional method comparison provided	Baseline not provided (focus on feature identification and interpretability)	Not reported
Ward et al. (2021)	Postoperative opioid use (adolescents)	Multiple ML algorithms	186,493 surgical encounters (ages 12–21)	Best algorithm AUC = 0.711 (significantly higher for certain surgeries)	No traditional risk model comparison provided	Baseline not provided (risk prediction focus, no PK comparator)	Not reported
Shen et al. (2025)	Voriconazole (<2 years old)	XGBoost	76 patients (110 TDM samples) + external validation	External validation *R* ^2^ = 0.75, RMSE = 0.14	PopPK model (NONMEM)	PopPK parameters used as inputs; SHAP identified CL, body weight, and laboratory values as important predictors	PopPK model (parameters as inputs)
Irie et al. (2025a)	Infliximab (pediatric Crohn’s)	XGBoost (ensemble of synthetic + real-world data)	Synthetic data (from published pediatric PK model, N = 560,000) + real-world validation (N = 292)	Initial RMSE = 6.44, MPE = 1.84; after ensemble, 5-fold CV comparable to Bayesian method	Bayesian estimation	Ensemble achieved comparable performance to Bayesian method (RMSE and MPE significantly improved)	Bayesian estimation
Fox et al. (2020)	T1D	RL	N = 30	Decreasing average risk by an additional 24% as meals increase in predictability	Basal-bolus (BB), PID control, and PID with meal announcements (PID-MA)	Leading to a decrease in median glycemic risk of nearly 50% from 8.34 to 4.24 and a decrease in total time hypoglycemic of 99.8%, from 4,610 days to 6	Basal-bolus (BB), PID control, and PID with meal announcements (PID-MA)

## 2 Reinforcement learning and closed-loop adaptive control of treatment timing

### 2.1 Reinforcement learning as a framework for sequential dosing decisions

The main value of reinforcement learning for clinical timing is that it reframes dosing as a chain of linked choices. This is not just a new tool. It changes the question we ask. We no longer treat a dose and its interval as one-off events. Instead, we see them as steps in a long sequence. Each step affects the next. The entire sequence determines the patient’s final outcome.

Clinicians face a problem regarding timing every time they prescribe and adjust a drug. A clinician must choose a dose amount and also choose when the next dose should be scheduled. A clinician makes these choices more than once because the patient’s pharmacokinetic (PK), PD, and clinical signs keep changing. This pattern makes dosing a step-by-step decision process instead of a single decision.

Reinforcement learning (RL) fits this pattern because RL is built for repeated choices over time. An RL system treats the patient’s current condition as a “state,” and it treats a dose choice and a timing choice as “actions.” The system then learns a rule for what to do next that aims to improve outcomes over the long run. This logic matters because one dose today can change the patient’s condition tomorrow, and that change can alter the later benefits and risks. RL keeps this time link at the center of the method, while a typical supervised model mainly learns fixed links between inputs and labels.

**FIGURE 2:**
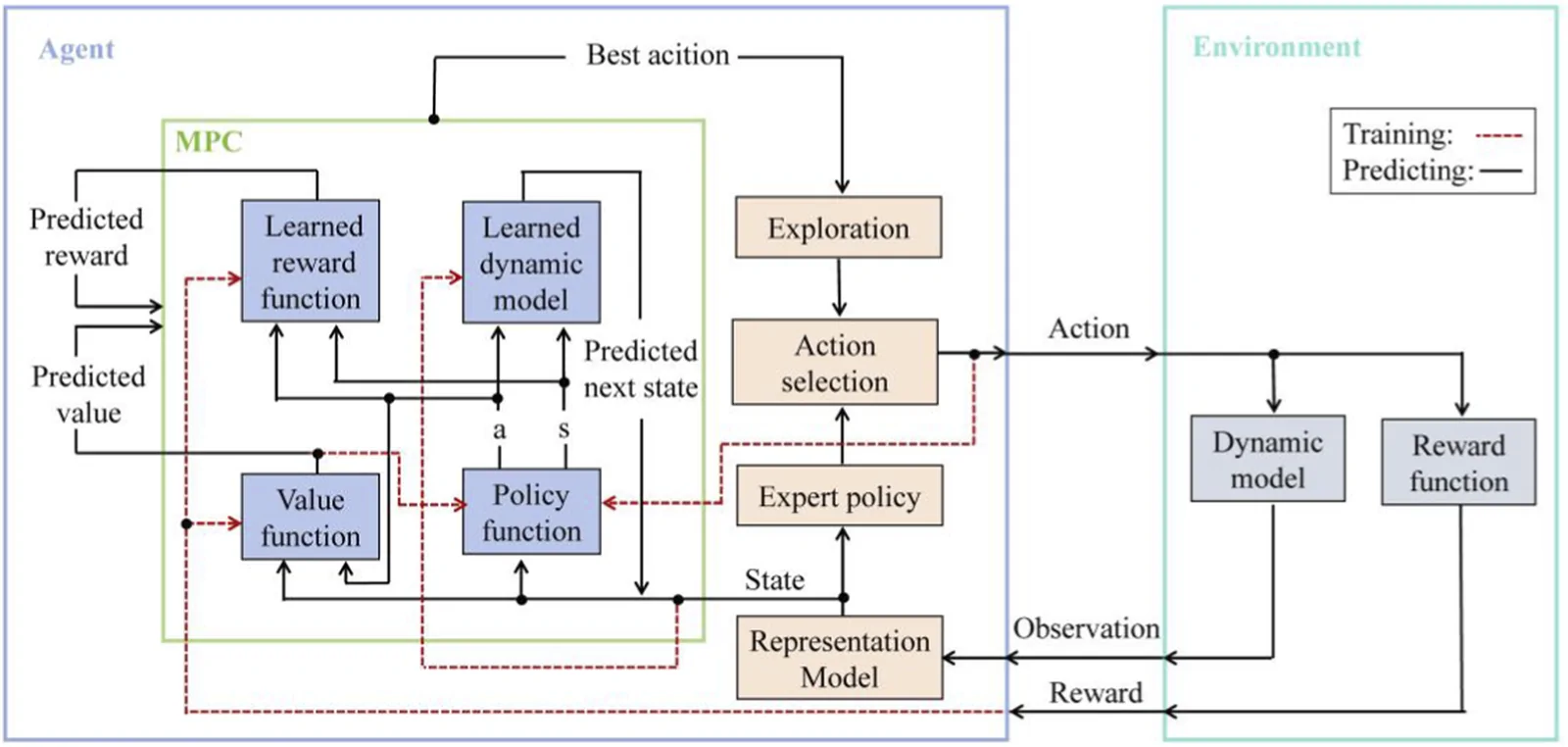
Reinforcement learning architecture diagram.

Some recent studies place RL inside mechanistic PK/PD simulators or “digital patient” setups. These virtual environments let an algorithm test long chains of dose and interval choices that would be impossible to fully test in real patients. The algorithm can then search for strategies that raise a reward score that combines benefit and safety. What these simulation studies show is that RL excels not at correctly guessing the next dose but at searching through many possible futures. It uses the PK/PD rules as a map. It then finds a full treatment path that meets long-term goals such as maintaining safe and effective drug levels over weeks or months. This line of work supports a simple idea. RL can serve as a practical engine for model-informed precision dosing (MIPD) because it optimizes a treatment path over time rather than predicting a single next step (De Carlo et al., 2025; Sabounchi et al., 2025; Tosca et al., 2024). Reinforcement learning is illustrated in Figure 2.

### 2.2 Personalizing dose and interval in chronic therapies with reinforcement learning

For chronic diseases, the real difficulty is how to keep a drug working without causing harm over many months. That balance is never still. It changes as the disease changes and as the patient’s body handles the drug differently. RL gives us a way to put a number on that balance. It does not treat every patient the same. It observes each person’s PK trends and biomarker readings. Then, it adjusts both the dose size and the gap between infusions over time.

Chronic diseases often force clinicians to adjust treatment slowly. A clinician may raise or lower dose levels. A clinician may also shorten or extend the time between infusions. These changes take place because the disease activity changes and the body’s handling of the drug can drift over months. RL can model this reality by using patient-level PK and biomarker trends as the “state” and by treating the dose size and interval length as choices that the system can control.

RL also has a useful feature for long-term care. An RL system can score today’s decision by the effect it causes later. A longer interval today can lead to a lower trough level later. A larger dose today can raise exposure and, later, the toxicity risk. RL can reflect these delayed effects by design because it links the value of an action to downstream outcomes and not just immediate results.

Pediatric Crohn’s disease provides a clear example. Irie et al. used a deep Q-network and paired it with an infliximab PK model. The system learned an infusion plan that chooses both the dose and interval. The interval remained within a clinically realistic range of 4–12 weeks, and the goal was to maintain the trough levels within a set therapeutic window while limiting overexposure (Irie et al., 2025b). The remarkable result was not that RL found one perfect interval. Rather, the RL policy gave each child a different interval. It based that choice on the child’s own clearance rate and inflammation markers. That is exactly what expert clinicians try to do, but the RL system does it with a fixed and repeatable rule. This approach also shows how RL can improve on a fixed schedule. A fixed Q8W plan gives the same spacing to everyone. Instead, the learned policy changes the interval based on each child’s predicted clearance and inflammation pattern, which mirrors what clinicians try to do but with more consistent rules.

Polycythemia vera shows another case with multiple targets at the same time. De Carlo et al. used Q-learning with a PK/PD model of givinostat and tracked several markers, including platelets, leukocytes, and hematocrit. The system produced dosing plans that adjust over time while balancing these markers together (De Carlo et al., 2025). Here, we see a clear conflict of timing. A doctor wants good control of the disease markers but also wants to avoid blood toxicity. Both these goals push in opposite directions. RL handles this by folding both into a single reward score. It does not need a simple linear trade-off. It can search for the best dynamic balance across time. This setting creates a real conflict of timing. A clinician wants strong control of the efficacy markers and additionally wants to avoid blood-related toxicity. RL can handle this kind of conflict because the reward can combine several goals in one score, and the method does not require the trade-offs to be simple or linear.

A broader message appears when researchers test RL as a general MIPD tool. A general RL-MIPD framework reported that policy learning lets an algorithm change the treatment intensity in response to patient-specific PK changes. The framework also emphasized that the system does not need to treat each visit to the clinic as an isolated choice (Van Den Berg et al., 2025). The key idea is continuity. A policy can use exposure history and current signals together, and it can choose the timing based on what it expects next.

These examples support one primary point. RL turns dosing into a moving control problem rather than a one-time calculation. The choice of timing of each dose depends on what has already happened, and each choice also aims to shape what happens next.

### 2.3 Machine learning for intermittent/pulsatile regimens and structured treatment interruptions

When we use intermittent schedules—with breaks in between—the hardest question is not whether to pause but exactly when to pause and when to restart. That is a timing-control problem. RL fits it naturally because RL was built for decisions that play out over time. Every “on” and every “off” changes the tumor’s behavior and the side effects seen in the patient. Thus, the timing of these switches becomes as important as the drug itself.

Clinicians do not always want a drug to run without breaks. Clinicians often consider intermittent or pulsatile schedules when side effects build up over time, when drug resistance starts to appear, or when a constant schedule becomes too difficult for a patient to maintain. In solid tumors, clinicians have used “drug holidays” as a practical tactic. Clinicians use these pauses to help patients tolerate treatment again. Clinicians also use pauses in hopes that the disease becomes easier to treat after the break. AI adds a more systematic approach here. AI can help a team decide *when* a pause should start and when treatment should restart, instead of relying only on trial-and-error timing.

RL fits this problem because RL makes decisions over time. Each dosing decision changes what happens next. Each choice can shift future tumor behavior and future toxicity. This structure makes intermittent control a natural match for RL. Researchers can train deep RL on mechanistic tumor models. The model can represent tumor growth and treatment response. The RL agent can then learn a pulsed pattern. The pattern can switch between high-intensity dosing periods and drug-free windows. The goal is to maintain stable control even when patients differ from each other (Eastman et al., 2021).

More complex PK/PD settings can also work with this idea. Actor–critic controllers can search for schedules on their own. These controllers do not need a person to hand-code a “break rule.” Breaks can appear as a learned strategy. This takes place when the reward function includes both toxicity costs and non-linear tumor responses. In such a setup, the algorithm has a clear reason to “back off” at some points (Mashayekhi et al., 2024). This tells us something important. A drug-free window is not a weakness or a compromise. Under the right biological conditions, it is the best strategy. When the reward function punishes long-term toxicity or resistance, the algorithm learns to step back at the right moments. This stepping back actually leads to better long-term control. Personalization strengthens the benefit. Deep Q-network (DQN) and deep deterministic policy gradient (DDPG) methods can adjust dosing cycles based on an individual tumor state. These adaptive cycles can beat fixed schedules or short-sighted schedules because the system can trade off control and exposure over time. The original text reports that these approaches can delay resistance and reduce the total exposure (Tordesillas and Arbelaiz, 2019).

Other frameworks support the same logic even when RL is not used. Evolutionary analysis and optimal control theory explain why intermittent adaptive therapy can outperform continuous dosing under selection pressure. This theory matters because it gives RL a reasoned foundation. It also helps explain why “on-off” timing is not just a convenience. It can be a control strategy.

Overall, these studies frame RL as a practical engine for finding safer interruption rhythms for each patient. The studies also shift intermittent dosing away from guesswork. The studies treat the timing of pauses as a high-precision control problem that uses data and mechanism-based signals.

### 2.4 Generative AI-assisted closed-loop systems: conceptual frontiers

Generative AI and closed-loop systems point toward a future that is not just about automating drug delivery. The bigger idea is a system that monitors the patient continuously. It predicts several steps ahead. It updates the timing plan in real time. This breaks the old habit of following a fixed calendar. Instead, the system responds to fast biological changes as they take place.

Some clinical problems change minute by minute. Early closed-loop AI systems focus on these fast shifts. Type 1 diabetes is a clear example. Deep RL-driven artificial pancreas systems can connect continuous glucose monitoring to automated insulin infusion. The system can then adjust the dose of insulin from one minute to the next. The original text reports that these systems can improve precision and increase time-in-range compared to that with traditional controllers (Fox et al., 2020). Safety-focused versions add guardrails. These versions can limit insulin delivery when hypoglycemia risk rises. This design teaches us a basic rule. A closed-loop system must set a clear boundary between being aggressive and being safe. It cannot chase the best possible control without limits. It has to respect safety at all times. It shows an important point. A closed-loop medical AI system can aim for strong control while also respecting strict safety needs (Zhao Y. F. et al., 2025). Reviews of automated insulin delivery also place deep RL as a key engine for future systems that learn personalized dosing rhythms across an entire day (Yu et al., 2025).

These ideas connect to oncology and other complex therapies. Digital-twin platforms can simulate a patient’s PK/PD path and tumor evolution. A team can use the simulation to test non-standard schedules before a real patient receives them. This step can reduce blind trial-and-error (Prunella et al., 2025). Generative AI can extend this setup. A virtual patient model could generate “what if” treatment scenarios. The model could estimate short-term biological changes under different timing choices. The model could then support real-time schedule updates. The original text describes a vision in which large language model-based systems combine multiple patient data streams, forecast tumor dynamics, and help clinicians make adaptive decisions (Derbal, 2024).

Together, these points describe a shift toward closed-loop systems that both predict and control. These systems would not follow a fixed calendar by default. These systems would respond to fast biological changes. These systems would also keep clinicians in the loop, especially when risk arises or when the data become uncertain.

### 2.5 Practical limitations and research gaps in RL-based dose-timing strategies

Before RL timing strategies can be used in routine care, we have to face several difficult problems. These problems are not just technical. They arise from the nature of RL itself. RL depends heavily on simulations. It needs a well-designed reward function. It learns by trying actions and observing what happens. All of these clash with clinical reality, where safety, clear explanations, and reliable performance are non-negotiable.

Many RL policies are trained in simulated settings. These settings often rely on PK/PD simulators. Clinical trust then depends on how well the simulator matches real patients. Real physiology can vary. Real patients can also behave in ways that the training data does not cover. Such mismatch can break a policy that appears strong in simulation (De Carlo et al., 2024; Irie et al., 2025b; Van Den Berg et al., 2025).

Reward design is another major problem. A reward function must match the clinical goals. A poorly chosen reward can push the algorithm toward schedules that look optimal mathematically but do not fit the clinical reality. This risk worsens when the task has many goals at once, such as tumor control, toxicity control, and patients’ quality of life.

Safety concerns also limit real-world exploration. RL often learns by trying actions and observing outcomes. Real patients cannot serve as a trial space for risky exploration. Thus, translation needs safeguards. The original text highlights off-policy evaluation, restricted action spaces, and conservative update rules as ways to reduce this risk.

Interpretability also remains difficult. A model can show which features matter, but the timing logic is still difficult to explain. A clinician may ask, “Why should the system pause *now*?” A sequential policy does not answer this question as easily as a simple risk score does.

To move forward, we need progress on many fronts at the same time. One fix here and another there will not be enough. Progress will require several steps. Researchers will need prospective trials. Researchers will also need validation that meets regulatory standards. Teams will likely need links to routine therapeutic drug monitoring. The link would let RL adapt to real drug concentration paths instead of only simulated ones (Shen et al., 2022; De Carlo et al., 2024; Irie et al., 2025b).

## 3 The application of artificial intelligence in treatment duration, regimen switching, and interval extension

### 3.1 Timing decisions beyond dosing: treatment duration and interval as machine learning targets

How long a patient stays on a drug and how often they take it are not minor details. They are major decisions that can be optimized just like the dose itself. AI and quantitative pharmacology let us treat time as a variable that we can learn from data and not a fixed rule we follow by habit.

Clinicians make timing choices that go far beyond picking a dose. A clinician also chooses how long a patient stays on therapy. A clinician also chooses how often a patient takes the drug. A clinician may also need a plan when a patient misses or delays a dose.

Many patients miss doses in routine care. These delays can lower the drug exposure. This problem can be especially serious in long-term neurological and infectious diseases. Researchers have started to study these timing disruptions with quantitative methods instead of only relying on general advice. Xie et al. used PopPK modeling with Monte Carlo simulation for pregabalin in epilepsy. The study compared different “rescue” options after missed or late doses. The study then offered numbers that can guide salvage dosing when adherence is poor (Xie et al., 2024).

Critically ill patients face a similar timing problem, even when clinicians administer the drug in a hospital. A clinician often needs to choose the best dosing interval. The interval can change whether the drug levels reach the targets and stay there. PopPK modeling and simulation studies on ceftriaxone, meropenem, and piperacillin have shown how the interval choices link to target attainment and clinical outcomes (O’Jeanson et al., 2021; Butragueño-Laiseca et al., 2022; Tang Girdwood et al., 2022).

AI further develops the idea by treating time as something a system can adjust. In HIV infection, researchers used deep reinforcement learning (double deep Q-networks) to plan structured treatment interruptions. The model directly optimized when therapy should pause and when it should restart. The goal was to balance viral suppression with the patients’ quality of life (Yoon et al., 2024). In oncology, pharmacokinetic modeling of sub-cutaneous atezolizumab examined interval extension based on weight or exposure thresholds. The analysis showed that longer intervals could reduce treatment costs while maintaining effective exposure (Kicken et al., 2024). Across these examples, a shared theme emerges. The treatment duration and frequency should be flexible. They should adapt to the patient’s changing state. AI’s job is to uncover the predictable links between the timing choices and the patient’s outcomes. These examples show the same theme. A system can treat duration and spacing as decision variables and not fixed settings.

Overall, these studies treat “how long” and “how often” as the central targets. AI and quantitative pharmacology change timing from a rule-of-thumb into a learnable and personalized optimization problem.

### 3.2 Predicting personalized treatment duration and discontinuation rules

When should a doctor stop a drug, reduce it, or restart it? The answer depends on finding the point where continued therapy no longer provides more benefit than risk. We can estimate that point using exposure–response data and biomarker trends. This turns a vague clinical judgment into a more quantitative prediction.

Clinicians often need to decide when a patient should stop, pause, or restart therapy. These decisions can shape long-term control. Many decisions still rely on experience because the best point to stop at is not always obvious.

Quantitative models now support more tailored duration decisions in several settings. In spinal muscular atrophy, PopPK simulation work for nusinersen sodium built re-initiation regimens after interruptions of different lengths. The model linked the interruption duration to the loading strategy needed to restore therapeutic cerebrospinal fluid concentrations (MacCannell et al., 2022). In psychiatric maintenance therapy, an exposure–response analysis for monthly aripiprazole connected the plasma concentration thresholds with relapse risk. The analysis provided a quantitative basis for deciding how long maintenance should continue and when tapering or discontinuation might be considered (Wang X. et al., 2025).

Some approaches combine learning methods with drug and effect models. De Carlo applied reinforcement learning with PK/PD modeling to givinostat therapy for polycythemia vera. The study produced adaptive dosing rules that adjust schedules and the overall duration to achieve sustained hematological response (De Carlo et al., 2025). In infectious diseases, PopPK and Monte Carlo simulation analyses have identified exposure targets related to efficacy, such as f%T>MIC. Researchers then used those targets to simulate optimized regimens for imipenem and cefazolin. This approach links duration decisions to microbiology and renal function conditions instead of using a fixed course length for every patient (Duke et al., 2024; Rao et al., 2025).

Taken together, these studies move duration decisions toward patient-specific rules. These rules rely on measured exposure and outcome relationships. They also support a clearer risk–benefit logic when clinicians consider stopping or continuing therapy.

### 3.3 Predicting the success of reducing the dosing frequency in maintenance therapy

When considering about administering a drug less often, we should not ask “what does the label say?” We should ask “does this patient’s PK and target occupancy allow a longer gap?” This is a quantitative question. We can answer it by comparing the exposure levels across different intervals. If the exposure stays equivalent, the longer interval can be safe.

After the disease control improves, clinicians often want fewer doses. A longer interval can reduce the burden for the patient. A longer interval can also limit ongoing toxicity when long exposure matters.

Several studies support interval extension across different fields. In oncology, retrospective cohort analyses compared pembrolizumab every 6 weeks with every 3 weeks in patients with non-small-cell lung cancer and breast cancer. The studies reported similar survival and safety profiles between schedules, which supports the practical use of longer intervals in real-world care (Jones et al., 2022; LeVee et al., 2025). In infectious disease prophylaxis, pharmacokinetic parametric modeling evaluated high-dose sub-cutaneous benzathine penicillin G. The modeling indicated that an interval could extend to 13 weeks while still meeting the target concentrations (Kado et al., 2023).

Pharmacokinetic simulation also explains *why* an interval can stretch without losing exposure. A model-informed drug development strategy designed alternative tislelizumab schedules every 2, 4, or 6 weeks. The design aimed to match exposure across schedules so the treatment could be more flexible without sacrificing efficacy (Rizwan et al., 2025). The key insight from this “exposure matching” is simple. Extending an interval means providing the same total exposure over a longer time. PK simulation lets us check, for each patient, whether that equivalence holds before we change the schedule. Population PK simulations in psychiatry make a similar point for long-acting injectable antipsychotics. These simulations indicate that some risperidone and aripiprazole formulations can maintain therapeutic plasma concentrations and dopamine D2 receptor occupancy with intervals of up to 2 months (Fagiolini et al., 2025; Perlstein et al., 2025; Snoeck et al., 2025).

In short, AI-supported PK analyses can help identify who can safely move to fewer doses. This shift reframes maintenance therapy. The clinician no longer asks only “what is the standard schedule?” The clinician also asks “which interval fits this patient safely?”

### 3.4 Using real-world machine learning to interpret and support interval regimen selection

Real-world machine learning does more than pick the best regimen. It reveals patterns in how clinicians actually choose intervals. It makes this hidden reasoning visible. We can then measure it, repeat it, and share it. That provides us more consistent and transparent decisions about dosing intervals.

Clinicians often choose among several approved regimens. The choice usually depends on more than the label. The clinician considers the patient factors, side effects, and patterns of use over time.

Real-world machine learning can make these timing patterns visible and measurable. One machine learning analysis of adverse event reports and medication patterns found that time-related features affect the reporting quality and treatment pathways (Choo et al., 2024). Another study used unsupervised machine learning with timing, dose, and concomitant use to group complex dosing behaviors. This grouping can improve exposure stratification in pharmacoepidemiology because it turns messy real-world use into clearer categories (Laksafoss et al., 2025).

Some studies also connect interpretable machine learning with PopPK to support regimen selection in high-risk groups. Shen et al. combined XGBoost with PopPK modeling and SHAP attribution to find key predictors of voriconazole exposure in infants. This approach supported data-driven choices for the initial dose and dosing interval (Shen et al., 2025). What makes this study useful is that SHAP attribution shows clinicians which features—such as the weight, age, or organ function–—drive the interval suggestion. This builds trust. The clinician can see why the model recommends a certain interval and not just what the recommendation is. Other tools translate PK models into simple risk scores that clinicians can use. MeroRisk is one such example that turns model outputs into a predictive score for target attainment under standard regimens (Liebchen et al., 2021).

Overall, real-world machine learning performs two functions. It describes how dosing intervals actually vary in practice. It also turns hidden clinical reasoning into steps that others can review and repeat. This change supports more consistent and transparent interval decisions.

### 3.5 Imaging-based AI for predicting tolerance to extended dosing intervals

Imaging gives us a window into whether a disease is truly stable. Structural and functional biomarkers from images can tell us if a patient is likely to handle a longer gap between doses. This lets us predict tolerance before we actually change the schedule.

Imaging-based care often gives clinicians an early signal about whether a disease is steady. Structural biomarkers can show what the tissue looks like. Functional biomarkers can show how the tissue acts. These two types of markers can help a team judge whether a patient might handle a longer gap between doses.

Researchers also use machine learning to scan and summarize research papers, not only in eye care but in other imaging-driven treatments. One text-mining study of photobiomodulation therapy literature pointed to a clear need for treatment plans that match each person. Previti et al. also emphasized that images and related signals can produce features that help guide how often treatment takes place and how long it continues (Previti et al., 2025). The study reinforces a useful idea. Imaging features can inform timing decisions early, before problems show up. They are not just for explaining the bad outcomes after they occur. This finding supports the idea that imaging and signal features can inform the dosing timing before problems appear.

Another line of study links imaging to dosing decisions through models of how drugs move through the body. Researchers combined physiologically based pharmacokinetic modeling with PET/CT imaging in order to study timing. This work showed how timing choices and add-on interventions can change tracer uptake at the tissue level. The same study kept tumor targeting intact while it explained these timing effects (Siebinga et al., 2023). This combination gives a clear reason to use imaging results when a team adjusts the timing for an individual patient.

Imaging AI, therefore, supports a second route to timing decisions. The approach uses structural and functional biomarkers as inputs. The approach aims to predict whether a patient can tolerate an extended dosing interval before the clinician changes the schedule.

### 3.6 Optimizing absolute therapeutic windows with causal machine learning

In acute care, timing is often a difficult constraint. A delay can reduce the benefit. An unnecessary extension can cause harm. Causal machine learning helps us move beyond “relative timing”—when to start or stop—to finding an “absolute window” where the benefit peaks and harm is minimized. This window is important because missing it can have lasting consequences.

Acute care often turns timing into a strict constraint. A delay can reduce benefit. An unnecessary extension can increase harm. Researchers use causal machine learning and related methods to find windows where timing matters the most.

Ward et al. examined postoperative opioid prescriptions. The analysis identified predictors of prolonged opioid use. The results can support earlier action when a patient shows signs of risk, so the care team can avoid continuing opioids longer than needed (Ward et al., 2021). This example links timing to duration decisions after surgery.

Another study used reinforcement learning to plan the timing and intensity for supportive treatment after surgery. Sabounchi et al. applied reinforcement learning to postoperative hemodynamic management. The model learned dosing choices for fluid and vasopressor infusion during the first 72 h after cardiac surgery. The study reported a decrease in persistent acute kidney injury (Sabounchi et al., 2025). This study shows that timing in postoperative care is a series of hourly decisions and not a single start time. Each hour’s fluid and vasopressor choice adds up over 72 h. The cumulative effect determines the kidney outcomes. This result frames timing as a sequence of decisions and not a single start time.

A third example is seen in HIV therapy. A concentration–response analysis linked drug exposure during treatment intervals to long-term metabolic outcomes. The analysis highlighted that both the start of therapy and the choice to continue therapy can shape later outcomes (Griesel et al., 2022). This point again places timing at the center of risk and benefit.

These studies support a shared conclusion. Causal learning and reinforcement learning can enhance timing work beyond “relative timing” and into absolute therapeutic windows. These windows are important because delays or overly long use can lead to consequences that may not be reversible.

### 3.7 Limitations and gaps in AI-driven decisions regarding treatment duration and interval

AI tools for treatment duration and interval have made real progress. However, they still face three big barriers before they can be implemented in everyday clinical procedures. First, they often fail when moved to a new hospital. Second, they rarely include exposure–response mechanisms. Third, we have very few prospective trials to test them. All three need barriers need to be overcome.

Researchers have to test whether the timing models work outside the place where they were built. External validation studies show that this step is difficult. Some PopPK and machine learning models lose accuracy when teams move them to new hospitals. Studies of posaconazole and ceftriaxone models showed weaker prediction after being transferred to new centers. These results point to over-fitting risk and poor transport across sites (Huang et al., 2022; Schouwenburg et al., 2025). This problem is significant because timing tools only help if they remain reliable in real clinical settings.

Expert reviews also show that practice varies widely even when tools exist. One consensus review discussed dosing and timing diversity for ATG/ATLG in transplantation. Battipaglia et al. (2025) showed that differences across teams make standardization difficult, even with quantitative support. This kind of variation can limit model training and model adoption at the same time.

Theme 3 also faces limitations in the types of data that feed the timing predictors. Many predictors for interval extension come from *post hoc* trial work or from imaging cohorts in a single center. These sources increase the risk of dataset shift and the risk of performance estimates that look better than they will look in a new population (Moosavi et al., 2021; Weidinger et al., 2025). A separate problem arises from real-world data. Real-world interval choices can reflect disease severity or resource limits, not only the biology. This mismatch can confound models that try to learn the “best” intervals from observed practice (Vora et al., 2024).

Another gap involves how models represent drug exposure. Many predictions about duration and intervals still do not include explicit exposure–response models. This gap is important because timing decisions always involve a trade-off. A clinician wants enough exposure for effect, and a clinician also wants to avoid toxic exposure (Tang et al., 2023; Gérard et al., 2025; Han et al., 2025; Irie et al., 2025a).

Going forward, we need stronger connections between prediction and mechanism. We should combine the predictors from this section with PK/PD constraints and step-by-step optimization tools such as RL from Section 2. This would create a closed loop: predict, optimize, and then validate. This design can support closed-loop validation. The central question is practical and direct. The question asks whether extended intervals can remain safe over the long term (De Carlo et al., 2025; Irie et al., 2025b; Van Den Berg et al., 2025). Clinical testing also remains limited. Prospective trials that randomize patients to AI-suggested timing strategies are still rare. These trials are important for regulatory support, especially when timing decisions carry high risk, such as antithrombotic duration or acute hemostatic intervention (Lee et al., 2024; Osawa et al., 2025).

Theme 3, therefore, shows both progress and unfinished work. AI already supports decisions about the treatment length, stopping time, and which patients may handle longer intervals. Theme 3 also shows that the field still needs prospective validation and stronger links to exposure-aware adaptive methods.

## 4 AI-driven chronotherapy and timed drug-delivery systems

### 4.1 Chronotherapy: synchronizing treatment with circadian biology via artificial intelligence

**FIGURE 3:**
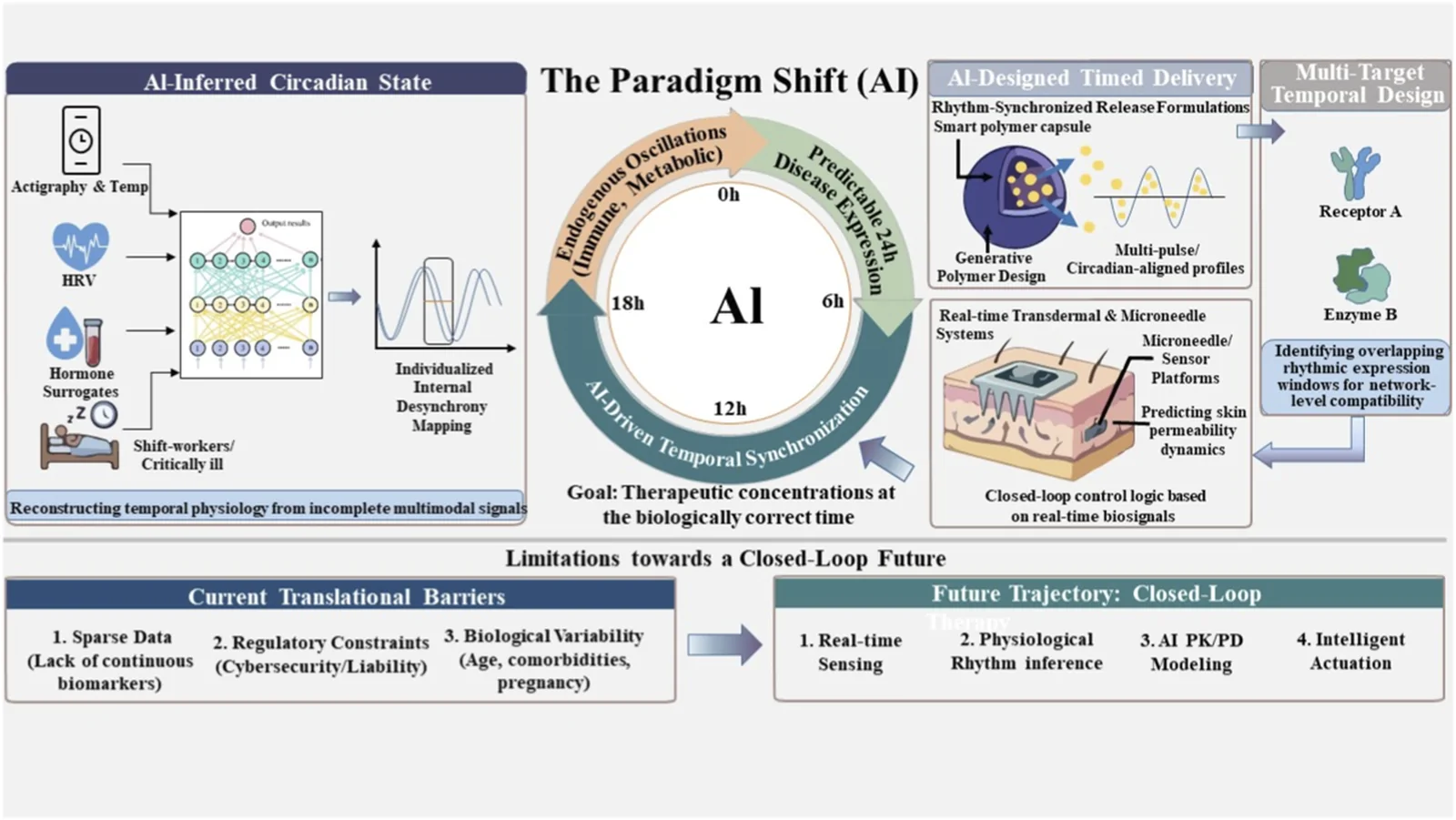
Use of AI algorithms in chronotherapy and timed drug delivery.

Figure 3 summarizes the application of AI algorithms in this field under the Theme 4 framework, along with some control feedback mechanisms within the drug metabolism and transport framework.

The basic idea of chronotherapy is simple. The effects of drugs are not the same at every hour of the day. They rise and fall with our internal 24-h cycle. Thus, the goal is not to administer a drug at a fixed clock time but to match the drug’s arrival to the time when the body responds the best. AI can help us find that time, even from incomplete data.

The human body runs on a daily cycle that repeats approximately every 24 h. This cycle acts like an internal schedule. The schedule helps control when the body releases hormones. The schedule also affects the immune activity, how the body uses energy, and how strongly blood vessels stay constricted. These changes do not stay constant from morning to night. Instead, the changes rise and fall across the day. As a result, symptoms and drug responses can also change in a pattern that often repeats each day.

Clinicians often notice these day–night changes during routine care. Many patients with asthma report worse symptoms close to dawn. These patients may wake up with restricted breathing at that time. Many patients with rheumatoid arthritis also report that mornings feel harder. In these patients, inflammation can be stronger earlier in the day. Some evidence links this morning pattern with higher morning IL-6 levels. Hypertension can also show a weaker drop in blood pressure at night, which clinicians often expect to see in healthier patterns. Fixed-time dosing often ignores these daily changes, so it can miss moments when the body is most sensitive or least responsive (Liu et al., 2022; Kole et al., 2025).

Artificial intelligence offers a different path because it can estimate a person’s internal “body time” even when the data are incomplete. A system can learn from mixed signals such as actigraphy (movement from wearables), skin temperature, heart rate variability, or hormone-related proxy markers. The system can then infer a likely circadian phase and flag when the internal clock is out of sync with the outside schedule. This is a big step forward. Without needing blood tests for melatonin or cortisol, AI can reconstruct a person’s circadian phase from wearable sensors. That makes chronotherapy practical in everyday clinics and not just in research labs. This approach is significant for shift workers, critically ill patients, and people with disrupted sleep–wake patterns because their rhythms often shift or break apart. These models can also connect timing to drug exposure, which helps explain why pharmacokinetic patterns vary across the day.

Several examples in the text tie this idea to specific modeling work. A population PK/PD model of linezolid in critically ill patients supports the view that time-linked physiology can shape drug behavior (Qin et al., 2025). A machine learning system that predicts doses for adolescent sertraline follows the same basic idea. The system uses patterns in data to support the dose choices. The system also targets a group whose bodies can change quickly as they grow and develop (Fu et al., 2024). This kind of setting makes fixed “one-size” dosing more difficult because the same dose can lead to different drug levels as the patient’s biology shifts. AI-linked population pharmacokinetic modeling for infant voriconazole pushes this time-aware thinking even earlier in life. The approach uses a model that can combine patient data with expected drug movement in the body. The approach also fits infants, whose daily rhythms and organ function can differ from adults (Shen et al., 2025). This difference is important because organs that clear drugs do not always behave like adult organs, and the change can affect exposure even over short periods. Putting these examples together, we see a clear pattern. Age, illness, and daily rhythms all create a “temporal context” for how the body handles a drug. AI’s job is to read that context and pick the best dosing time within it. Together, these two examples point to one clear message. A patient’s day-to-day physiology can change how the body handles a drug. A dosing plan should, therefore, match the time and changing biology instead of assuming that the body stays the same all day and across development.

The same timing problem becomes sharper in groups with major biological shifts. Pregnancy and childhood can change drug processing, and organ dysfunction can shift clearance in ways that may also vary with time. The text highlights levetiracetam in pregnancy as an example (Li et al., 2023). Schagen et al. (2023) also highlighted tacrolimus dosing algorithms that differ across circadian windows, which directly links dose decisions to time-of-day rules. These studies support a central theme in the original passage: AI can rebuild a patient’s time-based physiology, and the reconstruction can help clinicians aim for useful drug levels at biologically meaningful times.

### 4.2 AI-assisted design of multiphasic or circadian-synchronized release systems

When we design drug release systems for chronotherapy, we do not just want slow release. We want a release pattern that has multiple pulses—early and late—because it matches the body’s natural rhythms. AI helps us design the pulses and adjust them. This moves us from “release slowly” to “release in rhythm with the body.”

Chronotherapy often needs a drug to arrive in more than one wave. A therapy may need an early pulse and then a later pulse. This pattern can match daily changes in inflammatory cytokines, metabolic enzymes, autonomic activity, or hormone release. Traditional formulations usually follow a more fixed release curve, and such a curve may not line up with these rhythms. AI-driven design aims to make the release schedule more programmable and more aligned with daily biology (Kole et al., 2025).The text describes several building blocks that support this change. One approach uses generative design to propose polymer designs that match a chosen daily release plan. Another approach uses faster “stand-in” machine learning models to connect polymer ingredients and outside conditions to predicted release timing. A third approach uses closed-loop logic, where a system adjusts the pulse features based on real-time signals such as body temperature changes, glucose swings, or cytokine surges. These pieces describe a practical workflow: a system designs, predicts, and then adjusts.

Population PK/PD modeling in the text adds a clinical reason to take timing into consideration. A personalized TXA dosing framework for cardiac surgery highlights why infusion protocols may need adjustment during surgery as the physiology changes in real time (Liu et al., 2025). Exposure–response modeling for trofinetide links the treatment effect to time-related patterns rather than treating time as irrelevant (Darwish et al., 2024). For long-acting injections such as BUP-XR and aripiprazole, optimized PK models show that we should not assume a constant release rate. Metabolic clearance changes during the day. Therefore, the release profile should also vary to match those changes. “Long-acting” does not mean “flat;” it means “rhythm-aware.” Optimized population PK models for BUP-XR and aripiprazole long-acting injections also support the idea that clinicians can tailor long-acting schedules to daily changes in metabolic clearance (Wang et al., 2024). In combination, this evidence supports the text’s main pivot: the goal can move from “release slowly” to “release in step with the body.”

### 4.3 AI-driven transdermal and micro-needle systems for real-time timed delivery

Transdermal systems—patches and micro-needles—have a special advantage for timed delivery. The skin is easy to reach. We can apply sensors and delivery mechanisms together on one patch. This allows continuous sensing and adjustment. However, the skin is not stable. Hydration, temperature, and micro-channel openness all change with time. Therefore, the delivery system must adapt in real time and not follow a fixed curve.

Transdermal drug-delivery systems can support time-based dosing because they deliver drugs through the skin. These systems include micro-needle patches, materials that respond to electric signals, and sensor-plus-delivery designs. These systems also avoid first-pass metabolism in the liver, which can simplify some timing problems. A clinician or a device can also tune the drug flow more continuously because the delivery site stays accessible (Sabbagh et al., 2025). Machine learning can strengthen skin-based drug delivery because the model can estimate how quickly a drug moves through the skin. However, there is a catch. Skin hydration increases and decreases during the day. Micro-channels close or widen. Polymers swell. Humidity and temperature vary. All these changes alter how much drug gets through. So a pre-programmed release profile will not work. We need a system that measures the skin state and adjusts the release on the fly. Sabbagh et al. described skin delivery as a moving target and not a straight line. Skin hydration changes over time, and hydration can change how easily a drug passes through. The text also notes that micro-channels can change as time passes, so a pathway that looks “open” early may not stay the same later. The text adds that polymers can swell, and the swelling can change how the drug releases and spreads. The environment can add another layer of change. The text points to humidity and temperature as factors that can push skin conditions up or down. These conditions can also rise and fall during the day in patterns that look like a daily body rhythm. A simple fixed curve cannot easily follow all of these changes at once. The text, therefore, highlights hybrid simulators. These tools mix mechanism-based ideas with data-driven learning, so the simulator can reflect changing skin states instead of assuming one stable setting (Sabbagh et al., 2025).

Kim et al. then moved from skin delivery to dosing plans that deal with pharmacokinetic differences between patients. The text gives examples where an algorithm aims at a clear exposure target instead of using one default schedule for everyone. Cefoxitin target-controlled infusion (TCI) is an example. The text states that this approach can extend the time for which drug levels remain above the minimum inhibitory concentration (MIC) during surgery, which ties the infusion plan to a measurable goal (Kim et al., 2025). PBPK-optimized models for gentamicin in neonates and infants are another example. De Hoop-Sommen et al. (2023) explained that these models aim to place the peak and trough levels at specific times, so the plan focuses on “when” as much as on “how much.” Smit et al. also mentioned precision ibuprofen exposure modeling for patent ductus arteriosus treatment. The text uses this case to support the same theme: timing can shape effectiveness in a way that matters in real clinical care (Smit et al., 2023).

Finally, these research studies connect these timing-focused ideas to wearables. It describes a wearable platform as a steady data source for an AI model. The model can use these signals to anticipate daily changes, such as stronger inflammation in the morning or different autonomic activity at night. The system can then schedule drug release events to better match those expected shifts instead of releasing the drug at the same times every day.

### 4.4 AI multi-target design supporting chronotherapeutic strategies

When a drug acts on several targets at once, the timing becomes even trickier. Each target may have its own daily peak. One target might peak when another is low. AI can map these rhythms and find a time window where most targets align well. That helps us pick a dosing time that works for the whole network and not just for one part.

Multi-target drugs can face timing conflicts because different targets can follow different daily patterns. One target might peak when another target is low. This mismatch can weaken the overall effect if the dosing hits the wrong window. AI-integrated multi-target workflows aim to reduce this conflict by mapping how timing interacts with combined targets (Liu et al., 2022).

Lakkaraja et al. noted that the second uploaded file includes examples where machine learning-driven PK/PD modeling uncovers a hidden time structure in complex drug responses. Personalized rATG exposure modulation aims to time immune recovery after transplant, which treats timing as a part of the design goal (Lakkaraja et al., 2024). For nusinersen, the exposure–response model shows us that re-starting the drug should follow the natural rhythm of CSF metabolism. This is a reminder that even a drug with one main target still has to respect the body’s internal rhythms, because those rhythms affect how much of the drug reaches the target. Exposure–response modeling for nusinersen indicates that re-initiation schedules should match biologically set CSF metabolism rhythms, which again makes timing a core variable (MacCannell et al., 2022). PK variability of topiroxostat also shows how the enzyme rhythms and dosing time can interact, which can change what a “good” schedule looks like (Zhong et al., 2025). These examples support the passage’s larger idea: networks of targets can be rhythmic too, and AI can help identify time windows where multiple targets line up well enough to support effective combined action.

### 4.5 Limitations and future directions of AI-driven chronotherapy

AI chronotherapy sounds promising, but it faces real-world obstacles. Most patients do not wear continuous monitors. Good circadian markers such as melatonin require blood draws. Models trained in clean lab settings often fail in messy clinics. In addition, regulators worry about who is responsible if a system delivers a drug at the wrong time. These are not minor issues.

AI-based chronotherapy still faces practical barriers in real clinics. Many patients do not wear continuous monitors, so systems often lack dense time-series data. Strong circadian biomarkers such as melatonin and cortisol can also require invasive or specialized testing, which limits routine use. These shortcomings make it harder for models to estimate the circadian state reliably in everyday care.

Model performance can also decrease when models leave the lab. Models often learn from small or unusually clean PK/PD datasets, and those patterns may not transfer to messy real-world settings. The text points to real-world validation of isoniazid machine learning-guided dosing as an example where generalization becomes a challenge (Tang et al., 2024). The text also notes that population PK models can perform unevenly across settings, including cases such as vancomycin and linezolid.

Regulation adds another layer of friction. Autonomous delivery systems raise concerns about cybersecurity, data tracking, and who carries responsibility if a system releases a drug at the wrong time. Biology also adds complexity. Circadian variation can change with age, sex, other diseases, sleep disorders, and the environment. Studies in pregnancy, children, kidney disease, and critical care all show that circadian rhythms are not fixed. They vary as the patient’s condition changes. Therefore, a chronotherapy model cannot be set once and used forever. It must keep recalibrating as the patient’s state evolves.

Even with these limits, the direction in the original text stays consistent. AI-enabled chronotherapy is moving toward closed-loop, sensor-based, and adaptive treatment. This path combines real-time sensing, PK/PD modeling, rhythm estimation, and intelligent control. The result is not only patient-personalized dosing but also time-personalized dosing. The text frames this as a frontier with broad relevance for neurology, immunology, cardiometabolic disease, and oncology.

## 5 AI-enhanced precision dosing: the cornerstone of optimized therapeutic timing

Drug timing shapes whether a treatment works or whether it causes harm. A clinician must choose the dosing intervals, set infusion rates, and plan how long a treatment cycle runs. Traditional schedules often use fixed time gaps between doses. These fixed plans do not reflect how drug exposure and drug response can change across patients and within the same patient over time. AI is becoming a useful tool for this problem, especially when clinicians pair AI with pharmacokinetic ideas. AI can learn from sparse or complicated data. AI can also produce patient-level PK and PD predictions. These predictions give clinicians a practical way to build a dosing plan and then adjust the plan when the patient’s condition changes.

Some clinical areas make this need very clear. Neonatology, immunology, and critical care often show large changes in drug handling. Early exposure mistakes can also cause early harm in these settings. Studies on β-lactam and glycopeptide antibiotics show that even aggressive loading plans may not hit the early targets in patients with augmented renal clearance (Beijer et al., 2025). A separate model-based review of population-based meropenem guidance also shows major gaps between guideline dosing and patient needs (Raza et al., 2022). These examples point to one core issue. A system must predict exposure and then adapt fast when the physiology changes and clearance differs across patients. AI-enhanced PK modeling is designed to meet that demand.

**FIGURE 4:**
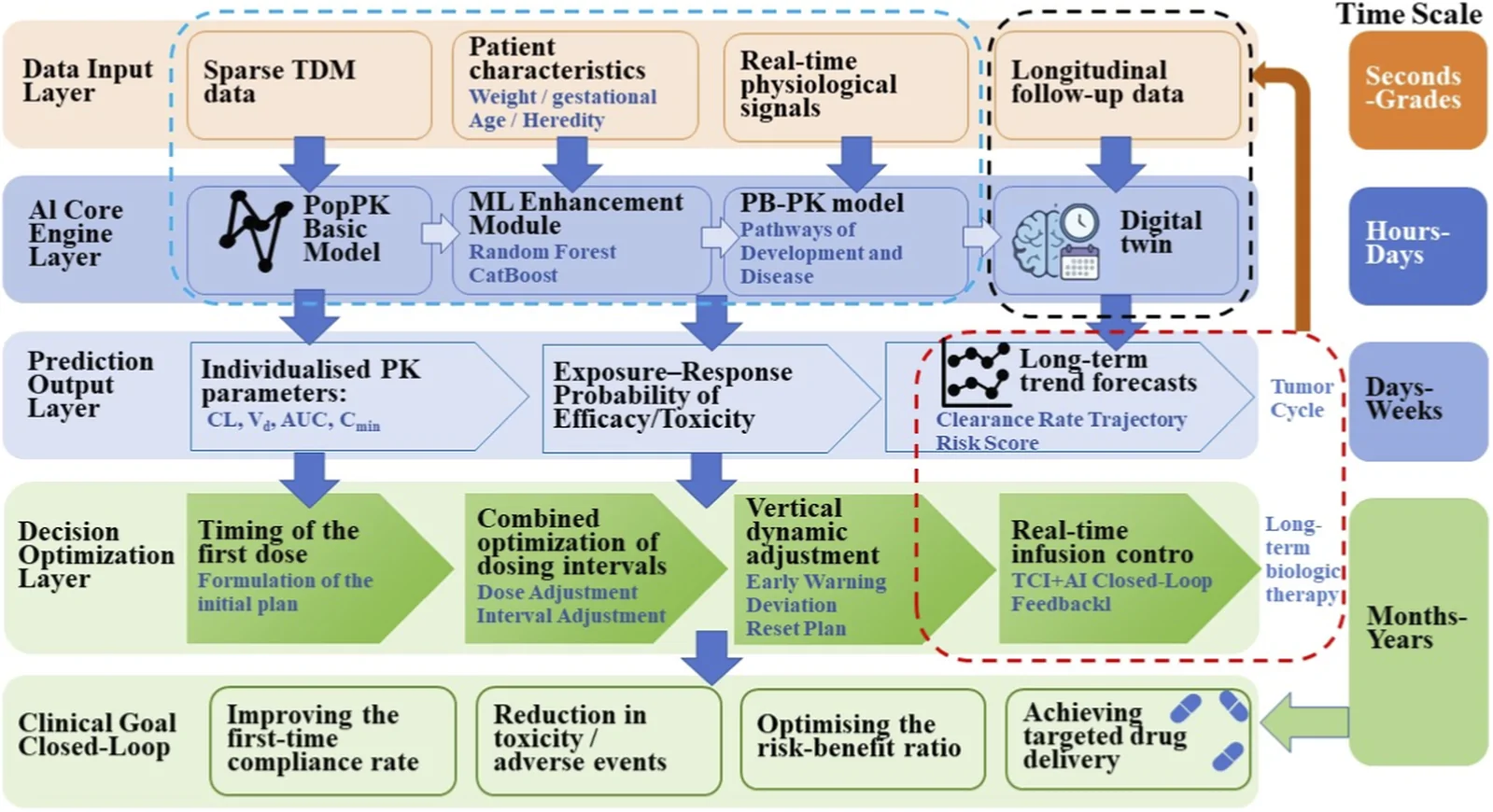
Use of AI algorithms in improving precision dosing.

Figure 4 shows the application of AI algorithms in areas related to improving precise governance, along with some control feedback mechanisms related to drug metabolism and transport.

### 5.1 The critical role of accurate exposure prediction for dosing timing

A clinician needs exposure–response knowledge to adjust a dosing interval or set treatment duration. A clinician also needs a clear view of how exposure changes over time. In practice, clinicians rarely collect frequent blood samples on a strict schedule. This limit makes it difficult to build a detailed PK curve in many real settings. This limit is more significant in neonates and in long-term disease care (Tang et al., 2023; Irie et al., 2025a; Kole et al., 2025).

Evidence from pediatrics and critical care points to a specific weak spot. Early dosing is often the point where targets are missed. Neonatal vancomycin therapy shows this issue. Standard population PK models produced early prospective predictions that diverged from observed exposure metrics in up to 60% of cases (Hughes et al., 2023). Critically ill pediatric patients on cefepime show a similar early risk. These patients can have changing renal function and fluid shifts. These changes can drive severe underexposure during the first dosing interval if a model does not account for them (Morales Junior et al., 2025). These findings support a clear message. Early exposure estimates are important, and steady-state estimates alone are not enough for timing decisions.

AI can reduce the gap created by limited sampling. Hybrid models that combine PopPK and ML show one practical route. The PopPK part provides a mechanism-based structure and known relationships. The ML part adds pattern learning that can capture complex changes and leftover variation that a strict parametric model may not capture (Gérard et al., 2025; Irie et al., 2025a). This pairing supports predictions of future exposure values from limited data. These predictions include outputs such as trough concentrations and AUC. The same pairing can also work with irregular or mixed real-world data. For example, ML tools can propose neonatal vancomycin regimens by using limited samples to predict exposure and then guide dose and interval choices (Tang et al., 2023).

Recent work also indicates that model design details can improve ML prediction. Ma et al. showed gains when ML models include PK structure terms as input features. In an elderly valproic acid cohort, a model that included PopPK-based clearance and volume terms improved the prediction performance, with an *R* ^2^ of 0.74 (Ma et al., 2024b). In ICU conditions, a combined PopPK-ML model for ticarcillin reached >76% accuracy in external validation (Ma et al., 2024c). PBPK approaches add another layer of timing support because they can simulate developmental and disease-linked paths over time. Studies show this support in cefotaxime dose conversion in neonates (Li Q. et al., 2024) and propylthiouracil population extrapolation (Shen et al., 2022).

Adaptive dosing depends on trustworthy exposure forecasts. AI-enhanced hybrid PK models aim to turn sparse and noisy clinical data into patient-level exposure predictions. These predictions provide the PK logic that clinicians need to set intervals, adjust duration, and refine timing rules. These models may improve further when they use real-time physiology streams and richer covariates. The original text does not specify which real-time signals are used, so that detail remains unknown.

### 5.2 Personalized dosing interval strategies via hybrid ML–PK models

A clinician must often adjust timing, not only the dose size. This point becomes obvious for drugs with a narrow therapeutic index such as vancomycin. A clinician faces risk on both sides. Underexposure can reduce the effect, and overexposure can increase the toxicity risk (Patanwala et al., 2022; Huang et al., 2025).

Many drug classes also show interval-linked outcomes. Antibiotics, immunosuppressants, and psychiatric drugs can all depend on timing choices. Isoniazid offers a clear timing marker. The 2-h post-dose level (C_2_h) links to safety and microbiological response, so the first interval choice is important. ML models that predict C_2_h can support interval selection more directly than a fixed schedule that assumes average behavior (Tang et al., 2024). Long-acting injectables also show timing effects. Aripiprazole exposure stability depends on interval settings, which shows that interval planning is important even when a drug is designed to last (Chepke et al., 2025).

Hybrid ML–PopPK methods target this interval problem by design. The PopPK part captures the main trend and known covariate links, such as weight and renal function. The ML part adds a second engine that can pick up patterns left in the model error. Some approaches use tools such as random forest or gradient boosting. These tools can reflect influences that the dataset does not measure directly, such as genetic, diet, or disease factors (Elias and König, 2025; Köllő et al., 2026). The result can be a patient-specific model that recommends a dose-and-interval pair to meet the target exposure goals. Integrated AI systems can also score candidate regimens and label them as “suitable” or “unsuitable” for a given patient, which can help with the first plan at the bedside (Ho et al., 2023).

Recent studies also support classification methods for interval-linked dosing decisions. In adolescents taking sertraline, a CatBoost model identified the appropriate dose strata, and this kind of stratification connects to the exposure range that an interval plan can achieve (Fu et al., 2024). Immunology work on IgG replacement therapy also highlights timing. PK simulations show that stable troughs depend more on interval tuning than on the total monthly dose alone (Li Z. et al., 2024). These findings reinforce the same theme across drug types: timing choices can be as central as the dose size.

Hybrid models blend mechanism-based structure with data-driven learning. They also push beyond “pick a dose for a standard interval.” They support joint planning, where the dose and interval function together as linked choices. This joint view sets the base for truly personalized timing strategies.

### 5.3 Exposure–response modeling for guiding longitudinal adjustments

A course of therapy usually unfolds over time. A patient’s response can change as weeks and months pass. A clinician often needs to change a dose or a dosing interval to match those changes. Each adjustment is also a timing choice. The clinician decides when to tighten control, when to step back, and when to recheck.

Some diseases and life stages make these timing choices harder because the body changes quickly. Pediatric immunosuppression can change as a child grows, and drug handling can also change along with that growth. Pregnancy-related epilepsy is another clear example because pregnancy changes physiology from one trimester to the next. Researchers have reported that levetiracetam clearance rises across pregnancy trimesters, so a clinician may need to review the dosing interval at each gestational stage instead of keeping one plan for the whole pregnancy (Li et al., 2023). Tuberculosis treatment can also require careful timing because efficacy and harm can change during long treatment courses. Linezolid in multidrug-resistant TB shows this problem in a practical way. Studies report clear differences in both toxicity and efficacy when clinicians compare twice-daily dosing with once-daily dosing (Li R. et al., 2024). These examples point to the same need. A team needs models that can anticipate PK/PD changes that depend on time and not just the patient’s identity.

Machine learning can help because it can link many inputs to one clinical outcome. Researchers can feed a model with PopPK-derived exposure measures and patient features, and the model can learn patterns that are hard to capture with simple rules. For example, teams can use methods such as random forest to find exposure cutoffs associated with benefit or harm, and they can estimate the chance that a specific patient will respond well or poorly (Han et al., 2025). This kind of work can support finding answers for decisions such as “Does this trough level look safe?” or “Does this exposure look high enough to work?” The model can then turn these patterns into a recommendation about whether the next adjustment should be performed immediately or later.

Clinical teams still need to understand why a model gives a recommendation. Many ML models act like a black box, so interpretability becomes a practical requirement for use at the bedside. SHAP (SHapley Additive exPlanations) is one approach that can break a prediction into parts. A SHAP report can show which inputs influenced the model the most toward a dose change recommendation, such as a specific trough value or a lab marker (Han et al., 2025). This kind of explanation is helpful because it gives clinicians a clear story for a timing decision, even when the underlying model is complex (Han et al., 2025).

Several examples show why timing models are important in real clinical follow-up. In phenobarbital therapy, PK parametric modeling found that a patient may need regimen changes based on inter-occasion variability rather than relying on fixed-dose steps each time (Stoschus et al., 2025). This result is significant because it indicates that the “same change” may not work at every visit, even for the same patient. In anticoagulation care, researchers trained time-series models on follow-up data and found that these models predicted bleeding risk better than static baseline predictors (Shahryari Fard et al., 2024). This comparison supports a simple idea. A model that tracks how a patient changes over time can guide interval decisions better than a model that only uses the starting conditions (Shahryari Fard et al., 2024).

AI-based exposure–response modeling can change long-term therapy planning. A clinician often reacts after a problem appears, such as breakthrough seizures, infection relapse, or early toxicity signs. A forecasting model can instead warn that a patient is drifting away from a safe and effective exposure range. The model can also suggest a patient-specific exposure goal, which helps a clinician decide both how to adjust and when to adjust. Over time, this approach can support decisions to shorten or extend the dosing intervals as the physiology and drug-response continue to change. The same logic aims to keep efficacy steady while lowering the chance of adverse events.

### 5.4 AI applications in infusion control and real-time anesthetic timing

Drug timing in healthcare does not follow one fixed schedule. A research program may take years to refine dosing rules and test models. A clinician in a procedure room must adjust therapy within minutes. A monitor can show meaningful changes within seconds. This wide range of time is significant because the patient’s condition can change quickly, and drug effects can follow these changes just as fast.

During medical procedures, a clinician often changes an infusion rate step by step. The clinician uses these minute-by-minute updates to keep the patient near a planned physiological target. Sedation depth is a clear example of such a target. The team aims to maintain sedation inside a safe zone rather than letting it swing up and down (Šafránková and Bruthans, 2025). A small dosing change can be significant because surgery stimulation and patient response can change from one minute to the next.

Critical illness creates another setting where timing becomes a life-or-death variable. A patient may need early antibiotics to control infection, and a patient may need early antifibrinolytic therapy to limit bleeding. Tranexamic acid in trauma care shows how strict the timing window can be. Evidence points to the best effect when clinicians administer the drug within 2 h after injury (Osawa et al., 2025). This point links dosing to accurate timing. A correct drug administered too late may not provide the same benefit.

Target-controlled infusion (TCI) offers a structured approach to this timing problem. A TCI system uses a dosing model to aim for a target drug level and then sets the infusion rates to reach it. New AI-enhanced control systems extend this approach. These systems add adaptive ML components or neural networks that read real-time biosignals, such as EEG and hemodynamics, and then adjust infusion decisions based on what the signals show (e.g., EEG and hemodynamics). This feedback design improves stability and helps the system avoid pushing the patient above or below the target range (Šafránková and Bruthans, 2025). It can also support smoother induction and emergence because the system can make frequent small corrections instead of waiting for a large drift to appear (Šafránková and Bruthans, 2025). Propofol TCI is a clear example. Its adaptive algorithms can respond to differences in patient sensitivity, with the goal of keeping cerebral drug concentrations steady across patients (Osawa et al., 2025).

Studies in perioperative pharmacology add more evidence that real-time dosing needs patient-specific thinking. PK modeling helps teams link infusion plans to how the body absorbs, distributes, and clears a drug. Recent PopPK studies on tranexamic acid indicate that a personalized infusion plan can retain the antifibrinolytic benefit while also lowering seizure risk (Liu et al., 2025). Early β-lactam therapy shows a different challenge. Drug levels after a loading dose can become unstable when renal function changes, and renal function can change quickly in sick patients. PK analysis in this setting indicates that only AI-enhanced or adaptive control systems can reliably maintain pathogen-suppressing concentrations when renal function is changing (Beijer et al., 2025). This detail explains why a fixed schedule may fail even when clinicians start with a strong initial dose.

At the shortest time scale, AI can act as an autopilot for infusion. The system can connect real-time patient response to drug delivery in a closed-loop pathway. The system can then shape the drug effect over time by updating decisions second by second. The same approach can also respond to early signs of physiological changes and adjust the dosing timing without waiting for the change to become obvious. This design points toward therapies that respond to the patient’s current state instead of following a rigid plan.

### 5.5 Digital twins and adaptive cycle control in oncology

Many cancer treatments follow a cyclic pattern. A clinic often sets the gap between cycles in advance. The fixed gap can shape outcomes. The gap is more significant when the drug is expensive or difficult for the body to tolerate, such as antibody–drug conjugates (ADCs) and immunotherapies (Lu et al., 2025; Monteiro et al., 2025). A short gap can enhance treatment intensity. A long gap can reduce pressure on the patient but may also change how well the therapy works.

Cycle timing also affects how the patient feels over time. A patient may face repeated fatigue, low blood counts, or other adverse effects that build across months of therapy. This timing issue does not appear only in oncology. IgG replacement therapy shows a clear timing signal in chronic care. Patients can see meaningful changes in trough levels when the interval extends beyond 4 weeks (Li Z. et al., 2024). This example supports a simple idea. A schedule that looks fine on a calendar can still create unstable exposure inside the body.

Digital twins offer a way to replace rigid schedules with flexible ones. A digital twin is a virtual model that represents a patient and updates over time. The model aims to match that patient’s disease course and drug response as new data arrive. AI-driven digital twins can combine several moving pieces, including changing clearance, rising or falling toxicity, and tumor biomarker response (Lu et al., 2025). The twin can then test “what-if” schedules in a simulated setting. The clinician can compare different cycle gaps without trying them on the real patient first.

This approach changes how a clinic chooses the next cycle date. A calendar-based plan sets the next visit early, often at the start of therapy. A twin-based plan can set the next visit later, after the system checks how the patient is doing. The twin can estimate when recovery is sufficient, when toxicity risk is the lowest, and when anti-tumor activity is likely to stay strong. This logic supports AI-guided dynamic scheduling, where the system keeps updating the timing choices as the patient’s biology changes (Monteiro et al., 2025). In this model, the “best day” for the next cycle is not a fixed day. The best day is a decision that the system revisits each cycle.

Time-series models support the same state-based mindset. Recurrent neural network models can learn patterns from repeated measurements over time. These models can map a patient’s changing risk as a trajectory instead of a single score. A study on sequential ML for dynamic prediction of bleeding risk in patients on anticoagulants shows that a model can update risk as new data arrive (Shahryari Fard et al., 2024). The example does not involve oncology, but the logic is similar. A system can adjust timing when the risk rises and relax timing when risk falls. Long-interval treatments also show how cycle planning can rely on modeling. Interval-adjusted biotherapies, such as 6-month paliperidone palmitate, show that long-cycle models can define treatment windows and guide when a clinician should re-check the patient (T’jollyn et al., 2024b).

Overall, AI-supported digital twins push cyclic therapy away from a strict calendar. Instead, the approach links timing to the patient state. The schedule can follow the patient’s own recovery pace rather than the clinic’s default interval. This change could improve the balance between benefit and harm for strong anticancer drugs, which is the core goal of a better therapeutic index. The same change will become more important as digital twins start to include toxicity recovery curves and PK/PD interactions. Interval personalization may then change from an optional feature to a central part of oncology treatment design.

### 5.6 ML-based PK prediction for biologics enables dosing regimen optimization

Biologic drugs often require dosing for long periods. Many patients receive monoclonal antibodies for months or years. This long timeline creates a practical problem. A patient’s pharmacokinetics can change over time, so one fixed schedule can stop fitting well.

Infliximab in pediatric inflammatory bowel disease (IBD) shows why a single interval can be limiting. Children grow, and growth can change how the body handles a drug. Some patients also develop immune responses to the drug, and the disease activity can change as well. These factors can change exposure during long-term therapy, so uniform dosing intervals can become a constraint (Irie et al., 2025a).

Several other biologics show the same pattern. Trastuzumab, paliperidone palmitate, intravenous immunoglobulin, and adalimumab all show a clear link between the dosing interval choices and how stable the trough concentrations remain. Trastuzumab maintenance provides a concrete example. Some patients could maintain disease control after an interval change from every 2 weeks to every 4 weeks, but only when they met specific week-16 criteria (Weidinger et al., 2025). Studies on adalimumab also highlight why interval choice is not one-size-fits-all. A PK similarity study that compared adalimumab with its biosimilar HS016 indicated several drivers at once. Body size, antibody formation, and clearance all acted together to shape an interval that suited a given patient (Zeng et al., 2022).

ML-based PK prediction offers a way to make interval decisions more patient-specific. Ensemble ML models can learn from both synthetic and real-world PK data. These models can predict future clearance and future trough concentrations with good accuracy (Irie et al., 2025a). This type of prediction supports a simple and practical workflow. A model can flag a patient who is likely to clear the drug quickly and fall to low trough concentrations. In that case, a shorter interval can better protect exposure. A model can also flag a patient with consistently high troughs. In that case, a longer interval can reduce the burden and cost while still maintaining exposure in a useful range. This approach is especially important in pediatrics because growth creates time-linked changes in PK, and those changes can appear gradually across a child’s life.

Another modeling study supports the idea that interval flexibility can be safe when the exposure target remains protected. Population simulations for 6-month paliperidone palmitate showed that a ± 2–3 weeks dosing window could keep drug exposure adequate, which adds clinically meaningful flexibility (T’jollyn et al., 2024b). PBPK analysis of IgG dosing also linked interval design to time-to-target behavior. The analysis showed how loading and maintenance intervals can change how fast a patient reaches a target trough concentration (Li et al., 2021). Together, these examples make a consistent point. Interval-focused PK prediction is a central tool for optimizing biologic regimens and not just a minor scheduling detail.

In chronic biologic therapy, AI can change the role of therapeutic drug monitoring. Traditional monitoring often behaves as a snapshot that triggers a one-time adjustment. A prediction-driven approach treats monitoring as an ongoing planning tool. The approach can shorten or lengthen dosing intervals over a patient’s course of life when PK changes. Biologics fit this strategy well because many have long half-lives and non-linear disposition, so interval changes can meaningfully shape exposure without requiring constant day-to-day changes in the dose.

### 5.7 Limitations and future directions

AI-guided timing shows clear promise, but several barriers still limit its routine use. Data quality remains a core issue. External validity also remains essential. A model can perform well with one dataset and still fail in another setting if the patient population demographic changes. Clinical practice also creates a data mismatch. Many ML methods work best with dense data; yet, real-world dosing often relies on sparse sampling (Tang et al., 2023; Gérard et al., 2025).

Even widely used drugs show these problems in concrete ways. Neonatal vancomycin models show performance fluctuations when the key inputs lack precision. Gestational age and creatinine are examples of inputs that can strongly affect predictions when they are measured or recorded with limited accuracy (Hughes et al., 2023). Linezolid modeling for multidrug-resistant TB shows another challenge. Multicenter PopPK evaluations found that the prediction accuracy can vary across sites, which reinforces the need for strong external validation before clinical rollout (Li R. et al., 2024).

Current hybrid approaches still leave room for improvement. Many systems mainly provide passive recommendations based on past data patterns. A system may suggest an adjustment, but the system may not truly optimize timing across the full course of illness. Future work may need other AI approaches to reach that level. RL is one such candidate. RL could help a system learn dosing policies through interactions with simulated or real patient pathways rather than only by copying historical patterns. Chronotherapy is another required link. Biological rhythms can change the drug response across the day; therefore, timing optimization should consider circadian and other repeating variables. Future models need to answer not only “how long” between doses but also “when” during the day should dosing occur to maximize benefit and reduce toxicity (Lu et al., 2025; Šafránková and Bruthans, 2025).

Better strategies for data collection can also strengthen timing models under real constraints. Bayesian limited sampling methods offer a clear example. A study on busulfan showed that targeted sampling plans can improve PK prediction accuracy even when full sampling is not realistic (Ben Hassine et al., 2024). This type of focused data-capture can support AI systems that need reliable inputs but must operate in real clinics.

Digital-twin methods may provide another step forward. A digital-twin approach can combine PK, PD, and biomarker data across different time scales. This integration can support simulation-based testing and real-time recalibration of dosing intervals as a patient’s state changes. This direction matches the goal of full temporal optimization because it treats timing as a moving target rather than a fixed rule.

AI has already built a strong base for personalized dosing timing through PK and exposure–response work. The next challenge is practical and technical at the same time. Researchers need to address the data limits, strengthen external validation, and design systems that learn and adapt. Researchers also need to integrate RL and chronobiology into the same timing framework. The long-term goal is an intelligent closed-loop approach that can adjust timing from very short to very long horizons. This combined path—machine learning, mechanistic modeling, and biological rhythm science—marks a key next frontier for precision dosing.

## 6 Future outlook

The previous sections show that artificial intelligence is changing how clinicians choose the timing of drug administration. AI can support decisions across several time scales, ranging from dose adjustments within one day to treatment changes over several weeks. However, technical progress alone cannot bring these tools into routine care. Clinical use depends on three connected areas: reliable data, strong external validation, and safe implementation. Progress in one area will remain limited unless the other areas improve at the same time.

### 6.1 Clinical data standardization and quality control

Reliable predictions begin with reliable clinical data. However, current healthcare data often contain large gaps. Clinicians may measure drug concentrations only when the treatment needs require testing. These samples do not usually follow a fixed schedule. As a result, AI models receive scattered measurements rather than the dense and regular data that many models expect. This problem is especially serious for newborns and critically ill patients because their physical condition can change quickly. Important patient variables also vary in quality. Hospitals may measure renal function at different intervals, while continuous circadian signals may not be recorded at all. These missing or uneven measurements add error to pharmacokinetic models. The error can then affect the estimates of drug exposure and recommended administration times. Training data create another concern. Some datasets include more patients from certain demographic groups than from others. A model may, therefore, perform well in its original sample but lose accuracy in a different patient population. Wearable devices could reduce some data gaps by recording continuous physiological signals. However, wearable data may contain movement artifacts, device-related differences, and private health information. Therefore, hospitals need common procedures for signal cleaning, device calibration, and privacy protection. They also need structured collection systems and shared data repositories with clear records of the origin of the data.

### 6.2 External validation and hybrid model generalizability

A model’s success in the hospital it is developed in does not prove that it will work elsewhere. External studies of vancomycin and linezolid models have shown this limitation. Single-center models may learn local treatment patterns instead of general clinical relationships. Differences in patient groups, laboratory methods, and prescribing habits can then reduce accuracy in another hospital. Model performance can also change over time. A patient’s physiology may change as the disease develops or treatment takes effect. The circadian phase can also alter drug handling during the day. A model that treats these relationships as fixed may become less useful as the patient’s condition changes. Hybrid models offer one possible response. These models combine mechanism-based PK/PD knowledge with machine learning. The PK/PD part keeps predictions connected to known biological processes. The machine learning part identifies patient-specific patterns that the fixed model may miss. However, each clinical setting may require a different balance between the two parts. Reinforcement learning creates an additional validation problem. A policy may appear safe in a simulator but behave differently in real patients whose responses fall outside the simulated assumptions. Therefore, researchers need careful testing before clinical use. The field also needs shared reporting standards. Common benchmarks for bias, precision, and clinical value would allow researchers to compare models directly and identify methods that generalize across hospitals.

### 6.3 Regulatory-compliant strategies and clinical translation

Clinical systems must include safety in the model design. Developers should limit the actions that an AI system can recommend. They should also set firm clinical constraints and allow clinicians to reject or change every recommendation. Closed-loop systems need added cybersecurity protection because software failures or outside attacks could directly affect treatment. Clinicians must also understand why a system recommends a timing change. Sequential AI policies are difficult to explain because each decision depends on earlier actions and patient responses. Limited transparency can reduce trust and make errors harder to detect. Regulators increasingly expect prospective trials that show meaningful patient benefits. Such trials are expensive, but adaptive designs and registry-based randomization may reduce the burden. Technical validation is also not enough. A useful tool must fit into the electronic health record, present information clearly, and support normal clinical work. Hospitals must train clinicians to interpret recommendations and respond when AI advice conflicts with professional judgment. Healthcare systems must also define responsibility before harm occurs because the liability for AI-supported decisions remains unclear.

### 6.4 Toward an integrated framework

Future systems should connect methods that currently operate separately. Reinforcement learning can guide a sequence of treatment decisions, while chronobiology can identify the best time of day for each decision. Digital twins could combine PK/PD models, disease progression, and sensor data. Researchers could then test timing strategies in a simulated patient before applying them in clinical care. Causal inference can also help researchers separate true timing effects from hidden influences. Together, these methods could move treatment away from fixed schedules. Future strategies may adjust drug timing according to the patient’s current physiological state and circadian phase. This transition will require continued investment in data systems, external testing, and implementation research. The effort is substantial, but safer and more effective treatment provides a strong reason to continue with the efforts.

## 7 Limitations

The systematic literature search for this study was limited to the PubMed database, which may introduce systematic search bias. The rationale for this limitation is that PubMed is a widely recognized core literature database in the biomedical field, covering high-quality journals most directly related to “the interdisciplinary application of AI in drug delivery timing,” such as those in clinical trials, pharmacology, and medical informatics. However, this topic is also highly interdisciplinary, and some literature involving algorithm design, sensor integration, and pharmacokinetic modeling is often first published in computer science, engineering, or chemistry databases (such as IEEE Xplore, Scopus, and arXiv) and specialized pharmaceutical sources. PubMed has limited coverage of discipline-specific conference papers, preprints, and regional journals, and it exhibits a bias toward English-language publications and update delays. Such systematic omissions caused by differences in database coverage are a major source of retrieval bias in systematic reviews and may lead to the omission of certain dosing algorithms, practices in non-English-speaking regions, or negative results in this review. Although the PRISMA guidelines recommend cross-validation across multiple databases, given the exploratory nature of this review and PubMed’s representativeness in biomedical informatics, we believe that a single database can still outline the core framework of this interdisciplinary field; however, the aforementioned risks of bias should be viewed with caution. When updating this review in the future, it will be necessary to conduct supplementary searches using Embase, Web of Science, and other databases in the field of computer science to improve the recall and the generalizability of the conclusions.
